# Tailoring Food Biopolymers into Biogels for Regenerative Wound Healing and Versatile Skin Bioelectronics

**DOI:** 10.1007/s40820-023-01099-1

**Published:** 2023-06-07

**Authors:** Qiankun Zeng, Qiwen Peng, Fangbing Wang, Guoyue Shi, Hossam Haick, Min Zhang

**Affiliations:** 1 School of Chemistry and Molecular Engineering, Shanghai Key Laboratory for Urban Ecological Processes and Eco-Restoration, East China Normal University, Shanghai, 200241 People’s Republic of China; 2 Department of Chemical Engineering and Russell Berrie Nanotechnology Institute, Technion - Israel Institute of Technology, 320003 Haifa, Israel

**Keywords:** Food biopolymers, Biogels, Skin bioelectronics, Deep wound, Superficial wound

## Abstract

An increasing utilization of wound-related therapeutic materials and skin bioelectronics urges the development of multifunctional biogels for personal therapy and health management. Nevertheless, conventional dressings and skin bioelectronics with single function, mechanical mismatches, and impracticality severely limit their widespread applications in clinical. Herein, we explore a gelling mechanism, fabrication method, and functionalization for broadly applicable food biopolymers-based biogels that unite the challenging needs of elastic yet injectable wound dressing and skin bioelectronics in a single system. We combine our biogels with functional nanomaterials, such as cuttlefish ink nanoparticles and silver nanowires, to endow the biogels with reactive oxygen species scavenging capacity and electrical conductivity, and finally realized the improvement in diabetic wound microenvironment and the monitoring of electrophysiological signals on skin. This line of research work sheds light on preparing food biopolymers-based biogels with multifunctional integration of wound treatment and smart medical treatment.

**Supplementary Information:**

The online version contains supplementary material available at 10.1007/s40820-023-01099-1.

## Highlights

- This food biopolymer-based biogel unites the challenging needs of elastic yet injectable wound dressing and skin bioelectronics in a single platform.
- This is the first demonstration of a hydrogel dressing that satisfies both deep and superficial wounds, and for the accelerated healing of diabetic wounds.
- Biogel-based flexible skin bioelectronic can serve as a “fever indicator” and monitoring human activities and tiny electrophysiological signals, providing important clinical information for the rehabilitation training of the wounded.

**Supplementary Information:**

The online version contains supplementary material available at 10.1007/s40820-023-01099-1.

## Graphical abstract

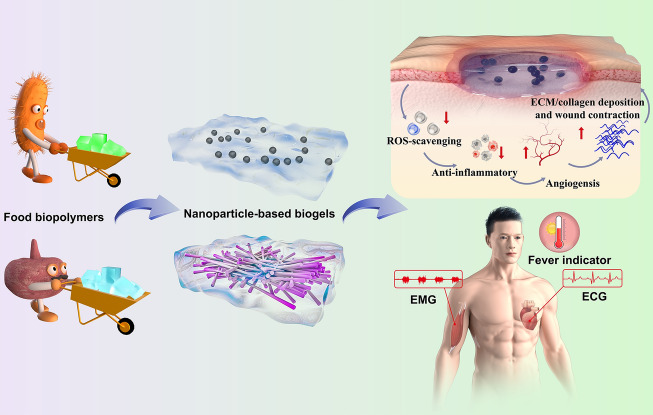

## Introduction

With the worldwide prevalence of diabetes, complications such as diabetic wounds have become a severe global issue, leading to poor life quality, high medical costs, and even amputation and death in patients [1–3]. One of the main causes for diabetic chronic wounds is the excess reactive oxygen species (ROS) generated by hyperglycemia-mediated overexpression of advanced glycation end products [4]. Excessive ROS accumulation on wound tissue has been confirmed to promote the expression of proinflammatory factors, restricted angiogenesis, and disrupted collagen deposition [5]. Scavenging ROS to improve the harsh wound microenvironment has become a critical strategy to promote tissue repair and regeneration in diabetic wounds [6]. In recent years, biocatalytic or antioxidant nanomaterials have enabled new ways of ROS scavenging and thus treating ROS-related diseases, such as polyphenol nanoparticles, redox polymer, platinum, ceria, and carbon [7]. However, the complex wound morphology in reality poses great challenges for the delivery of nanoparticles. On the one hand, deep wounds require nanocarriers with strong fluidity to fully fit the three-dimensional structure of the wound in an injectable manner [8, 9]. On the other hand, superficial wounds require nanocarriers to have good elasticity and shape recovery ability to withstand possible external extrusion. Unfortunately, few injectable and elastic carrier materials have yet been developed for diabetic wound therapy.

Besides the healing process, recent advances in skin bioelectronics can provide rich dynamic electrophysiological signals of the wounded, complementing traditional monitoring methods limited to wound sites [10, 11]. For example, elevated body temperature (> 2.2 °C) is a marker of infection and inflammation, which may be employed as an early predictor to standardize the assessment of chronic wounds [12]. Electrocardiogram (ECG) and electromyogram (EMG) assess the physiological state to assist the injured in rehabilitation training. In this endeavor, EMG signals collected by stretchable electrodes have already been used to control prosthetic limb systems for paralyzed patients and wheelchairs for patients with spinal cord injuries [13]. A major challenge for reliable epidermis–bioelectronics integration lies in the difficulty to develop an ideal electrode: 1) It is highly conductive to collect high-quality skin physiological signals and 2) it has mechanical softness matching that of the human epidermis to facilitate the collection and transmission of transdermal signals [14, 15]. Nevertheless, the large mechanical mismatch between traditional rigid skin bioelectronics and soft biosurfaces may lead to undesirable attenuation or loss of critical signals and even ultimately serious misdiagnosis [16, 17].

**Scheme 1:**
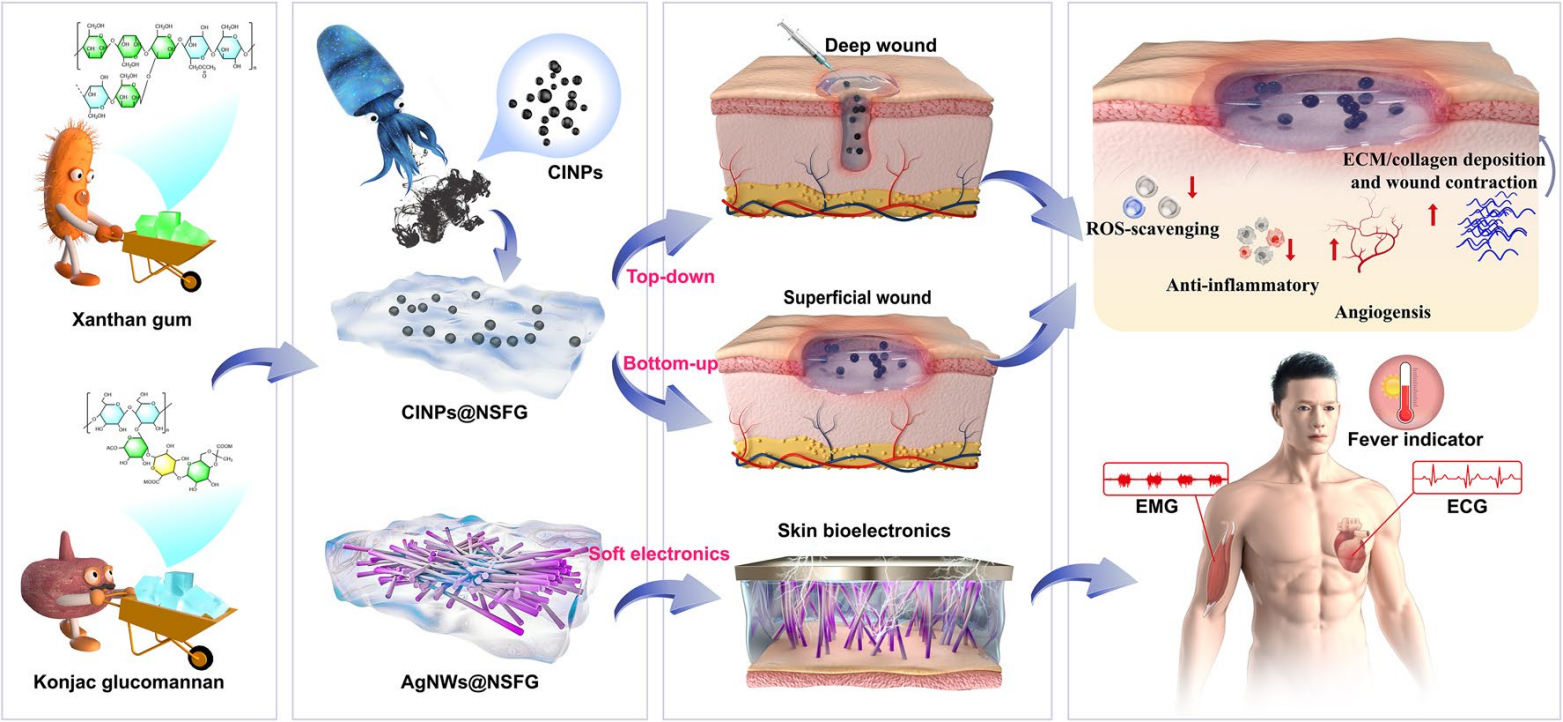
Schematic illustration of the preparation of food biopolymer-based biogels toward the development of antioxidative system for diabetic chronic wound healing and the unveiling of versatile skin bioelectronics

In this study, we explore a gelling mechanism, fabrication method and functionalization for a broadly applicable food biopolymers-based biogels that unite the challenging needs of elastic yet injectable wound dressings and skin bioelectronics in a single system (Scheme 1). Our naturally sourced food gels (NSFG) can be prepared from xanthan gum and konjac gum by bottom-up (self-assembly) and top-down (injectable) techniques as building blocks that are mechanically tunable, biodegradability, biocompatibility, low cost, and source renewability. In addition, the involvement of functional nanoparticles can broaden the utility of NSFG. Natural melanin nanoparticles extracted from cuttlefish ink (CINPs) serve as a tailor-made functional additive in NSFG due to the inherent biocompatibility and fascinating antioxidant effects [18]. We thus prepared a composite hydrogel of CINPs@NSFG with superior ROS scavenging capacity and systematically characterized and validated its diabetic wound healing efficacy in cell/animal models to optimize therapeutic workflow and provide potentially streamlined clinical translation. Subsequently, the introduction of the silver nanowires (AgNWs) network endowed the NSFG with excellent conductivity, making it possible to serve as flexible skin bioelectronics with rapid responsiveness, high sensitivity, and wide sensing range. The composite hydrogel of AgNWs@NSFG was then assembled into multifunctional skin biosensors for "fever indicator" and monitoring human activities and tiny electrophysiological signals (such as ECG and EMG), providing important clinical information for the rehabilitation training of the wounded. This line of research work sheds light on preparing food biopolymers-based biogels with multifunctional integration of wound treatment and smart medical treatment.

## Experimental Section

### Synthesis of Injectable Soft Biogel

The injectable soft biogel was prepared by a three-step method. In brief, the fully hydrated konjac glucomannan solution (1.0%) and xanthan gum solution (1.0%) were obtained by dissolving polysaccharide powder in deionized water with magnetic stirring at 80 °C for 1 h. The konjac glucomannan and xanthan gum solution was mixed in a volume ratio of 1:1 and vigorously stirred at 80 °C for 30 min. During the above process, the water lost due to heating was replenished with deionized water. Finally, the mixed solution was poured into a pre-prepared silicone mold and cooled to room temperature to form a composite gel network [19, 20].

### Preparation of CINPs@NSFG

The CINPs were detached from the ink sac of new cuttlefish by utilizing a straightforward differential centrifugation strategy. Briefly, the diluted cuttlefish juice was centrifuged at 1,800 rpm for 3 min to eliminate large precipitates. At last, the as-obtained CINPs were centrifuged at 8,000 rpm for 8 min and washed with deionized water for several times [21]. For the preparation of CINPs@NSFG hydrogel, CINPs at a final concentration of 1 mg mL^−1^ were added to a mixed solution of konjac glucomannan and xanthan gum, followed by programmed heating and cooling.

### Preparation of AgNWs@NSFG

The AgNWs were synthesized by a simple and effective polyol reduction strategies. In a typical synthesis procedure, 2.26 g of PVP was dissolved into 40 mL of ethylene glycol and heated to 160 °C until the temperature was steady. Then, 100 mL of FeCl_3_ solution (0.05 M) was added to the PVP solution followed by adding 100 mL of NaCl solution (0.15 M) and 10 mL AgNO_3_ solution (1.5 M). After 2 h, the reaction was quenched by adding deionized water and cooled to room temperature. The resulting AgNWs solution was purified three times with ethanol and water and finally dispersed in water [22]. For the preparation of AgNWs@NSFG, AgNWs at a final concentration of 1 mg mL^−1^ were added to a mixed solution of konjac glucomannan and xanthan gum, followed by programmed heating and cooling.

### Characterization of Samples

The morphology of samples was observed using a scanning electron microscope (SEM, Zeiss, Germany, Gemini 300) and a transmission electron microscope (TEM, Tecnai G2 F20). Fourier transform infrared (FTIR) spectra of samples were obtained by a Thermo Fisher Nicolet IS-10 spectrometer. Thermogravimetric analysis (TGA) of samples was carried out by a thermal analyzer (PerkinElmer, TGA 8000).

## Results and Discussion

### Synthesis and Characterization of Food Biopolymer-Based Biogels

**Fig. 1:**
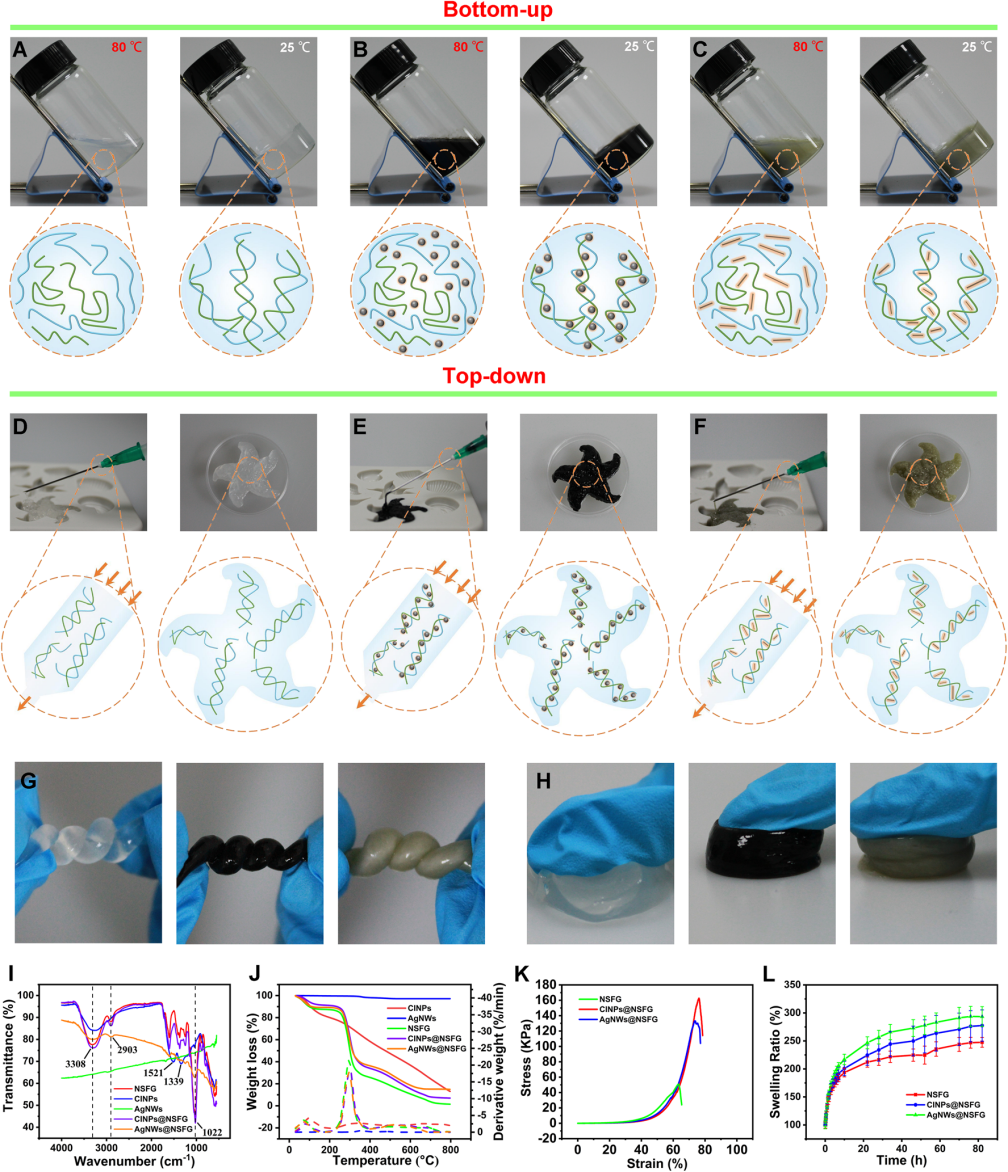
Preparation and characterization of biogels. Photographs and schematic representations of **A** NSFG, **B** CINPs@NSFG, and **C** AgNWs@ NSFG before and after gelation by the bottom-up approach. Photographs and schematic representations of **D** NSFG, **E** CINPs@NSFG, and **F** AgNWs@NSFG before and after injection via the top-down approach. Photographs of NSFG, CINPs@NSFG, and AgNWs@NSFG **G** withstanding distortion and **H** compression. **I** FTIR spectra and **J** TGA curve of CINPs, AgNWs, NSFG, CINPs@NSFG, and AgNWs@NSFG. **K** Compression curves and **L** swelling ratio of the biogels indicated (n = 3). The bar graphs represent mean ± SD

Xanthan gum is a microbial exopolysaccharide produced by *Xanthomonas campestris* using carbohydrates (such as corn starch) as the main raw material through fermentation engineering. It is a polysaccharide biopolymer consisting of D-glucose, D-mannose and D-glucuronic acid in a ratio of 2:2:1, with a relative molecular mass of more than 1 million (Fig. S1) [23]. The secondary structure of xanthan gum is that the side chains are reversely wound around the backbone of the main chain and are maintained by hydrogen bonds to form a rod-like double-helix structure [24]. Konjac glucomannan is derived from the tuber of the plant konjac and is a polysaccharide biopolymer composed of D-mannose and D-glucose units via β-1,4 linkage (Fig. S2) [25]. These two FDA-approved food biopolymers can exhibit remarkable synergistic gelation properties, forming elastic gels at a blending concentration of 1%. Its gelation ability is mainly attributed to the double-helix structure of xanthan gum molecule, which is easy to chimerize with β-1,4 linkage of polysaccharide molecules. When the temperature rises to 80 °C, the double-stranded xanthan gum molecules are activated, changing from an ordered state (helix structure) to a disordered state (random coil). When the temperature is lowered to room temperature, the xanthan gum and konjac gum molecules are fully chimerized together to form an elastic biogel (Fig. 1A). During the cooling process, the free NSFG and functional nanomaterials (e.g., CINPs or AgNWs) in solution were firmly embedded in the gel network and endowed the NSFG with new functions (Fig. 1B, C). This temperature-triggered sol-to-gel state transition physical gel can be synthesized by both bottom-up (Fig. 1A–C) and top-down (Fig. 1D–F) techniques [26]. In the bottom-up method, gels were constructed by physical cross-linking of polysaccharide molecules, whose shape and size can be controlled by specific molds. The obtained hydrogel has strong elasticity, can withstand large external pressure, and is suitable for superficial wounds that are often squeezed or deformed (Figs. 1G, H and S3). In the top-down approach, the gel is first performed in a syringe and extruded through a needle to fill the space of any three-dimensional shape (Fig. S4). The obtained hydrogel has strong shape adaptability, can be injected into any part of the body, and is suitable for deep wounds with irregular shapes. The chemical structures of NSFG, CINPs, AgNWs, CINPs@NSFG, and AgNWs@NSFG were confirmed by FTIR spectroscopy (Fig. 1J). The peaks at 3308, 2903, and 1022 cm^−1^ were attributed to O–H, C–H, and C–OH stretching of polysaccharides [27]. The consecutive aromatic bending (C = C, C = N) at 1521 cm^−1^ and carbonyl stretching (C = O) at 1339 cm^−1^ indicate that CINPs might be composed of peptides or proteins [28]. Moreover, no transmission peak was observed for AgNWs. TGA curves of NSFG and two composites of CINPs@NSFG and AgNWs@NSFG showed similar trends, decomposing at 220–380 °C (Fig. 1K).

Wounds and skin bioelectronics are often deformed by motion or external forces, so both strength and elasticity are crucial during hydrogel application [29]. Macroscopic compression experiments showed that the composite hydrogels can withstand compression without significant breakage, while NSFG was fragile and easily broken under the same pressure (Figs. 1H and S3). Subsequently, the mechanical strength of different hydrogels was quantified by rheological and compression experiments. The respective addition of CINPs and AgNWs to NSFG can enhance its mechanical properties (Fig. 1K), thus enabling the composite hydrogels to withstand higher stress (3.18 and 2.58 times) and strain (1.21 and 1.16 times). Similar results were obtained in rheological tests (Fig. S5). The free water contained in the NSFG network and the nanomaterial-enhanced NSFG network is the potential driving forces for the high elasticity and strength of composite hydrogels. On the one hand, the xanthan gum contained in NSFG is an extremely hydrophilic biopolymer that locks a lot of water in the NSFG network and prevents it from escaping during compression. On the other hand, the embellishment by CINPs and AgNWs can enhance the compression resistance of the inner wall of NSFG, preventing it from breaking under pressure. The microscopic mechanism of NSFG and two composite hydrogels during original, compression, and recovery is shown in Fig. S6. Upon challenged with external pressure, the dynamic water in NSFG conducts the pressure to the NSFG network and deforms it. Once the removal of the applied pressure, the nanomaterial-enhanced NSFG network can generate a reverse driving force, resulting in a rapid recovery of the entire structure. In the swelling test, NSFG and two composite hydrogels reached equilibrium after undergoing swelling for up to 70 h (Fig. 1L). An interesting phenomenon observed is that the doping of nanomaterials can significantly enhance the water absorption capacity of NSFG, which may be due to the enhancement of the NSFG network by nanomaterials to accommodate more water. This property would allow NSFG to absorb more wound exudate, thereby facilitating wound healing. Moreover, the doping of nanomaterials cannot significantly affect the water retention properties of NSFG (Fig. S7). Inspired by the above results, the doping of phenol red (PR, a pH-sensitive dye) to NSFG was also demonstrated to form a functional biogel of PR@NSFG, and the as-prepared PR@NSFG can be applied for the monitoring of wound pH (Fig. S8), which further verified the versatility of NSFG.

### In Vitro and in Vivo Biocompatibility

**Fig. 2:**
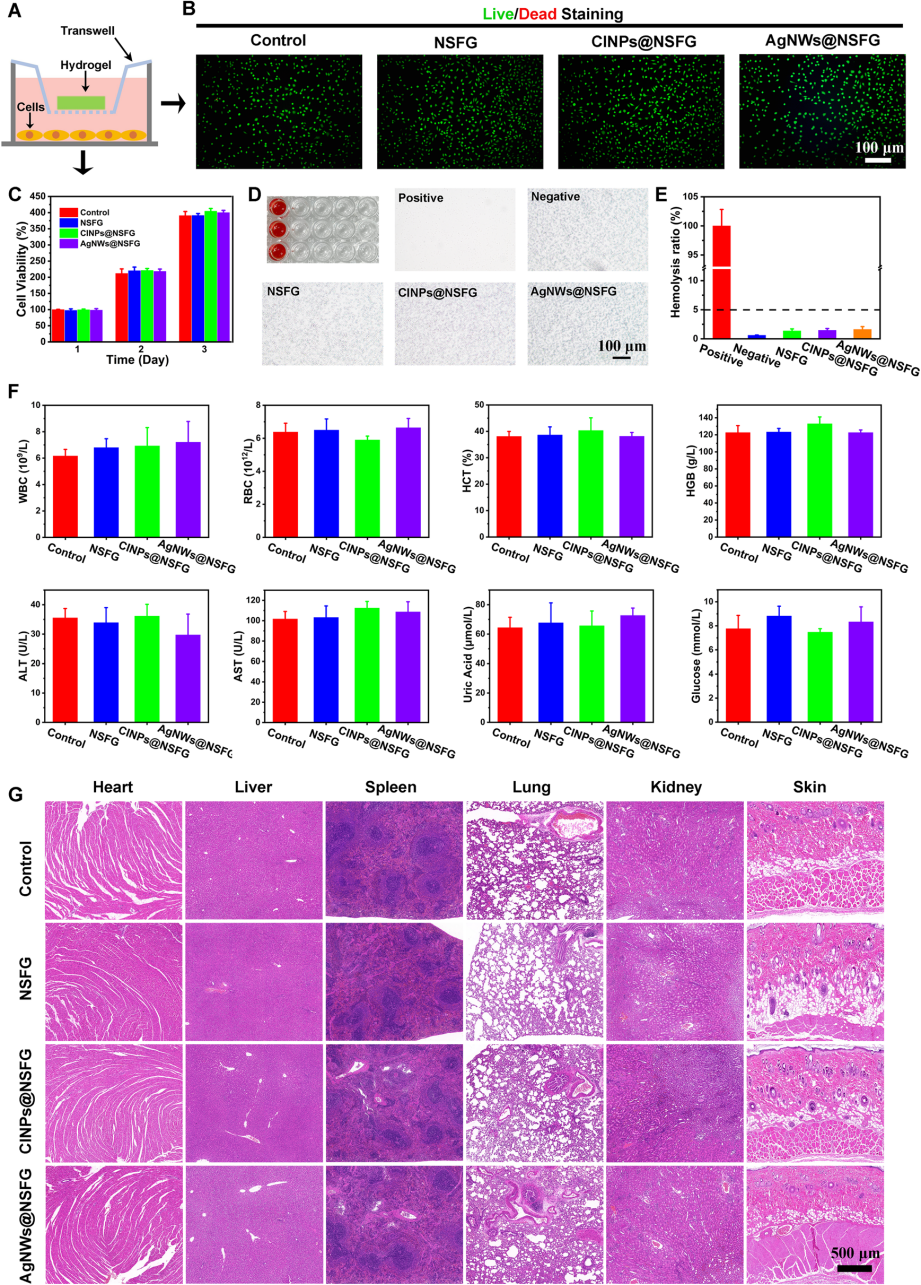
In vitro and in vivo biocompatibility. **A** Schematic diagram of cell–biogel co-culture model with the aid of transwell inserts. **B** Live/dead staining images of L929 after co-culture with various biogels. **C** CCK-8 assay of cell proliferation after 1, 2, and 3 days of co-cultured with various biogels (*n* = 3). **D** Photographs of the supernatant and blood cells after the biogels were incubated with blood at 37 °C for 1 h. **E** Quantitative statistical data for hemolysis ratios of various biogels (*n* = 3). **F** Blood biochemical tests of rats 10 days after subcutaneous injection of various biogels (*n* = 3). **G** Tissue staining (H&E) of major organs in rats 10 days after subcutaneous injection of various biogels. The bar graphs represent mean ± SD

Favorable biocompatibility is an essential requirement for biomaterials in medical applications as it will be in direct contact with tissues and blood for a long time [30]. Therefore, in vitro (cytocompatibility and hemocompatibility) and in vivo experiments were carried out to completely assess the biocompatibility of our biogels. Using the co-culture system of transwell inserts, live/dead staining and the cell counting kit (CCK-8) were performed to measure the effect of biogels of NSFG, CINPs@NSFG, and AgNWs@NSFG on the viability of L929 cells after co-culture (Fig. 2A). Gratifyingly, the results of live/dead staining demonstrated that almost all cells show normal spindle-like morphology and emit bright green signals, indicating that the biogels with high biocompatibility have no effect on the morphology and relative viability of L929 cells (Figs. 2B and S9). Subsequently, CCK-8 assay showed that after co-incubation of the biogels with L929 cells for 1, 2, and 3 days, there was no significant difference in cell number compared with the control group, indicating its excellent cytocompatibility (Fig. 2C). An ideal wound dressing should cause little or no hemolysis on the contact with a bleeding wound [31]. We explored the interaction between the biogels and blood components by co-incubating the biogels with PBS containing 5% v/v rat blood. After co-incubation at 37 °C for 1 h, the biogel group showed similar supernatant color and blood cell status to the negative control (Fig. 2D). Further calculations also confirmed that the hemolysis rate of all biogels was less than 5%, which is considered a safe level for biomedical materials (Fig. 2E). For in vivo biocompatibility testing, biogel samples were injected under the skin of rats for 10 days to evaluate their long-term biocompatibility. Thereafter, blood biochemical tests were performed on rats, including WBC (white blood cells), RBC (red blood cells), HCT (hematocrit), HGB (hemoglobin), ALT (alanine transaminase), AST (aspartate transaminase), uric acid, and glucose. There was no significant difference between the control and experimental groups, and their values remained within the normal range (Fig. 2F). At the same time, hematoxylin and eosin (H&E) staining was also performed on the tissues of representative organs (heart, liver, spleen, lung, kidney, and skin) to assess whether they had any damage, and no obvious inflammation and damage were observed. Overall, the biogels of NSFG, CINPs@NSFG, and AgNWs@NSFG exhibit excellent biocompatibility in vitro and in vivo, suggesting that they are promising candidates for wound healing and bioelectronic applications.

### Antioxidant Mechanism and ROS Scavenging Capacity

**Fig. 3:**
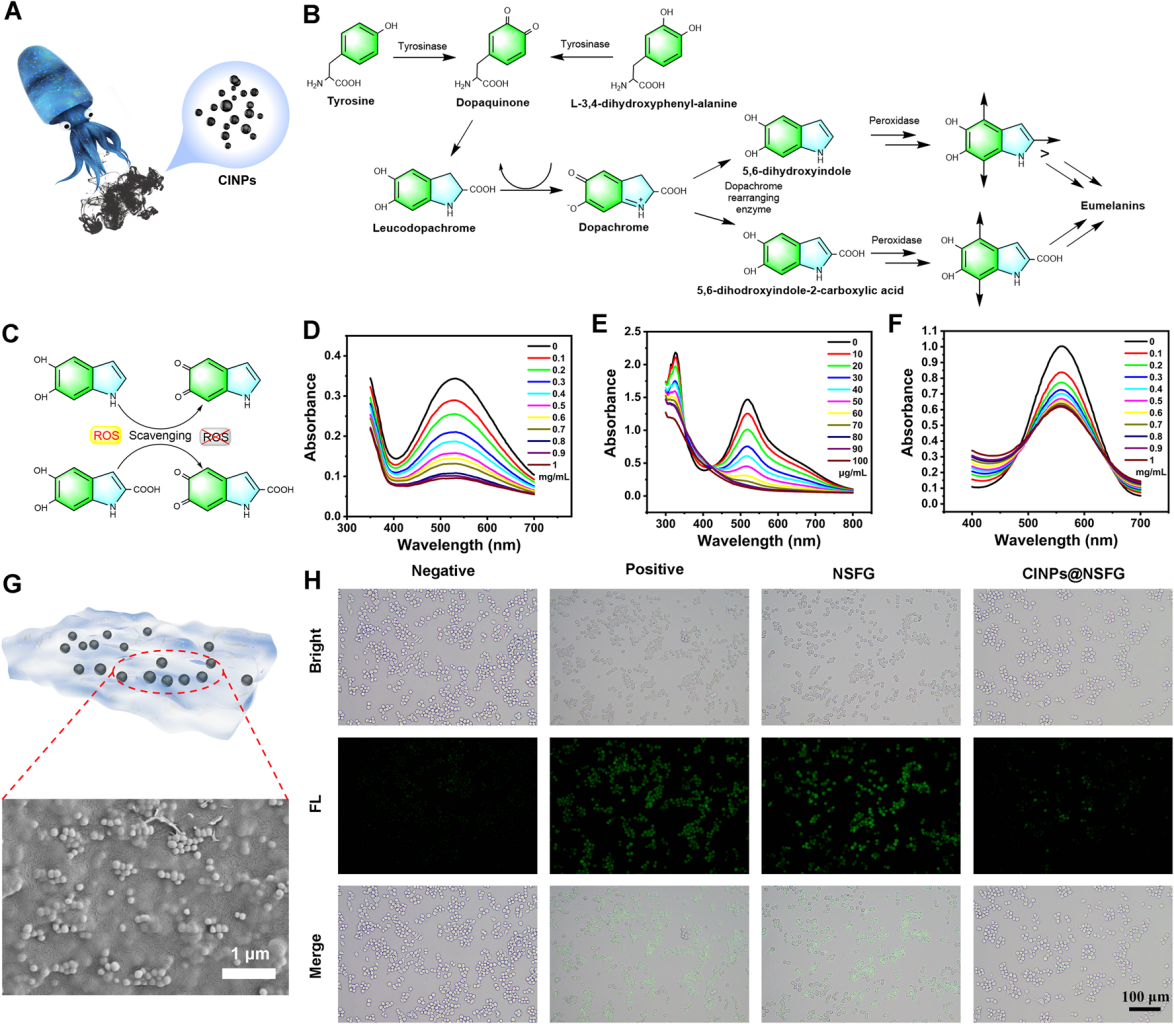
Antioxidative properties of CINPs and CINPs@NSFG. **A** Schematic diagram of cuttlefish juice containing CINPs. **B** Biochemical pathways of melanin production. **C** Antioxidative mechanism of CINPs. Full absorbance curves for **D** ·OH, **E** DPPH, and **F** PTIO scavenging assay. **G** Schematic and high-magnification micrograph of CINPs@NSFG. **H** Intracellular ROS scavenging performance of CINPs and CINPs@NSFG

In addition to inherent biocompatibility, CINPs also possess fascinating physicochemical properties, such as antioxidant capacity. The melanin with abundant reducing functional groups in CINPs is the guarantee that it can act as a ROS scavenger (Figs. 3A and S10) [32]. The biosynthesis of melanin in cuttlefish is a highly regulated process that begins with the conversion of the tyrosine and L-3,4-dihydroxyphenyl-alanine to dopaquinone by tyrosinase (Fig. 3B). Dopaquinone is unstable and non-enzymatically converted to dopachrome, which is then broken down to 5,6-dihydroxyindole or 5,6-dihodroxyindole-2-carboxylic acid by the action of dopachrome rearranging enzyme. Finally, it is polymerized into eumelanin under the catalysis of peroxidase specific to the ink gland. The antioxidant activity of melanin is closely related to its structure: the catechol group can act as a hydrogen donor and scavenge ROS to generate quinone groups (Fig. 3C) [33]. Besides, the two ortho-phenolic hydroxyl groups of the catechol group can chelate metal ions, thereby reducing the generation of ROS catalyzed by metal ions.

Having successfully understood the antioxidant mechanism of CINPs, we then verified their antioxidant capacity. ·OH is a typical oxygen radical, and its scavenging efficiency was evaluated by a salicylic acid probe with a characteristic absorption peak at 510 nm (Fig. S11A) [34]. In the presence of CINPs, the absorption peak near 510 nm in the UV absorption spectrum was significantly reduced, which confirmed that CINPs can effectively eliminate ·OH (Fig. 3D). Figure S12A shows the dependence of the ·OH scavenging efficiency on CINPs and increased with the content of CINPs. 1,1-Diphenyl-2-picryl-hydrazyl (DPPH) and 2-phenyl-4,4,5,5-tetramethylimidazoline-3-oxide-1-oxyl (PTIO) are two typical nitrogen free radicals, which are widely used to evaluate the antioxidant capacity of nanomaterials in vitro (Fig. S11B, C) [35]. As expected, CINPs had significant scavenging effects on both DPPH and PTIO in a concentration-dependent manner (Fig. 3E, F). Notably, CINPs were more efficient in scavenging DPPH (Fig. S12B, C). After treatment with 70 μg mL^−1^ CINPs, the absorption peak of DPPH at 517 nm almost disappeared, and the clearance rate reached more than 80%.

The successful validation of the antioxidant capacity of CINPs further inspired us to explore their application in conferring ROS scavenging capacity to NSFG (Figs. 3G and S13). The classical DCFH-DA fluorescent probe was used to measure the intracellular ROS levels of macrophages in the CINPs@NSFG groups with oxidative stress damage (Figs. 3H and S14). When macrophages were incubated in H_2_O_2_-containing medium, intense green fluorescence was generated, indicating successful induction of cellular oxidative stress (positive group). Notably, no significant change in the intensity of the green fluorescent signal was observed when cells were co-cultured with the NSFG. In sharp contrast, the fluorescence signal of the CINPs@NSFG group decreased to a similar level to the negative group, which proved that the antioxidant capacity of the CINPs@NSFG originated from the doping of CINPs. The above results confirmed our hypothesis that the doping of the corresponding functional nanomaterials could endow NSFG with specific properties, e.g., antioxidant capacity.

### In Vivo Diabetic Wound Healing Assessment

**Fig. 4:**
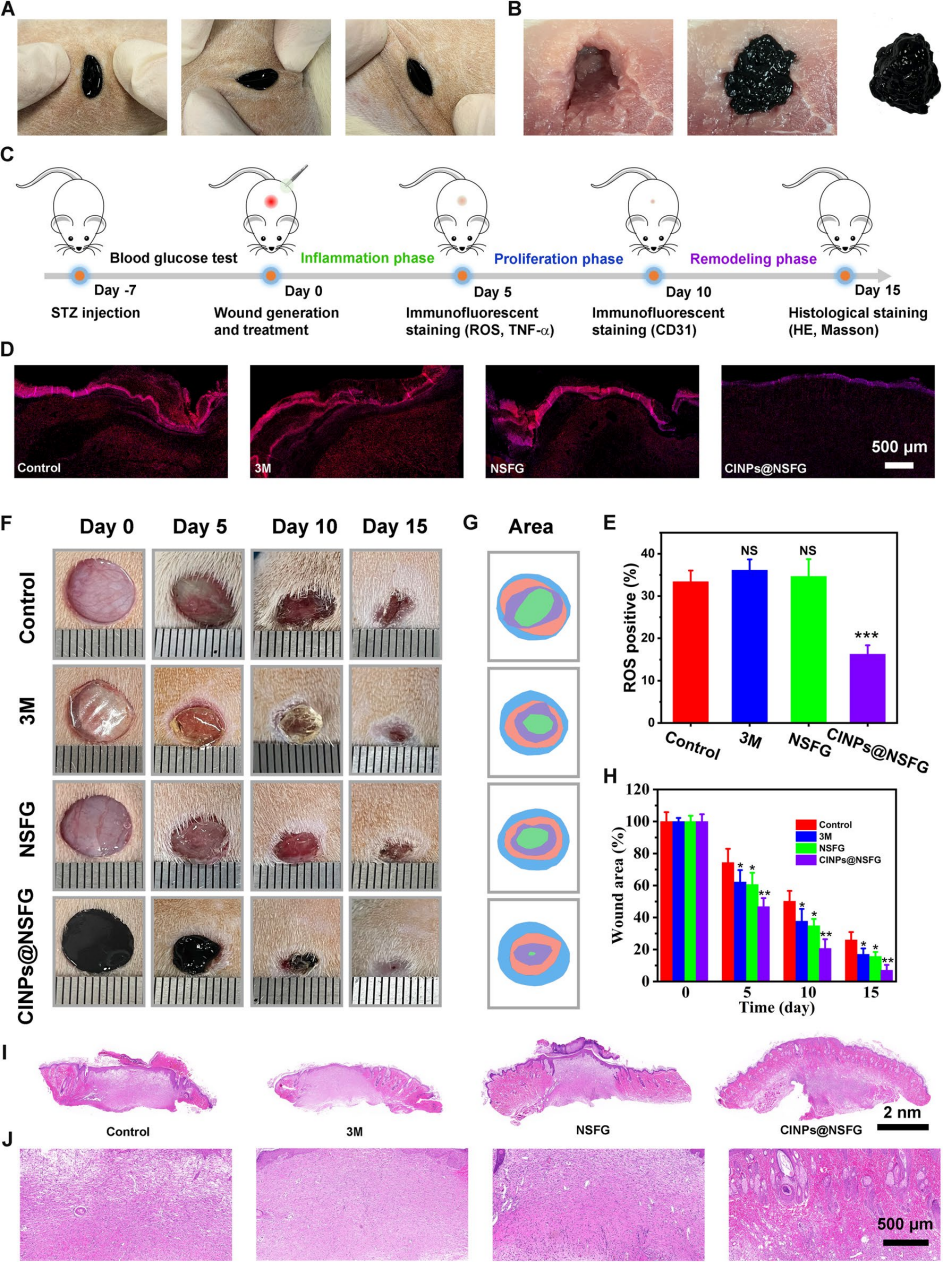
In vivo assessment of diabetic wound healing. **A** Photographs of the wound containing CINPs@NSFG subjected to compression and stretching. **B** Photographs of deep wound, wound filled with CINPs@NSFG, and detached CINPs@NSFG from the wound. **C** Diagram of the animal experiment procedure for testing the therapeutic efficacy of diabetic wound. **D** ROS staining of the wound on day 5. **E** Quantitative analysis of the relative coverage area of ROS in different groups (*n* = 5). **F** Photographs of the diabetic wounds with different treatments at representative time points post wounding. **G** Closure traces and **H** areas statistics of wound bed by different treatments at days 0, 5, 10, and 15 (*n* = 5). **I**, **J** Representative H&E staining of wound tissues at day 15. The bar graphs represent mean ± SD; *p* > 0.05 (NS: not significant); *p* < 0.05 (*); *p* < 0.01 (**); *p* < 0.001 (***)

In vitro experiments show that NSFG doped with CINPs (CINPs@NSFG) has both top-down and bottom-up delivery modes, which can overcome the narrow application range of traditional hydrogels. In this respect, we verified the practicability of CINPs@NSFG in rat superficial wounds and deep pork wounds, respectively. CINPs@NSFG with excellent elasticity were first synthesized using a bottom-up approach for filling rat superficial wounds (Fig. S15A). Then, the rat wound was repeatedly squeezed and stretched to simulate the external disturbances that skin wounds with hydrogel dressings may face in daily life (Fig. 4A). As shown in Fig. S15B, CINPs@NSFG can withstand strong external pressure without breaking and quickly recover to the original shape when the external force is removed. When applied to irregular deep wounds, most hydrogel dressings are impotent due to lack of shape adaptability. Thus, we employed a top-down synthesis of CINPs@NSFG in syringes and extrude it through needles to fill deep pork wounds with different shapes (Figs. 4B and S16). The results verify that our CINPs@NSFG can fill deep wounds with varied shapes and achieve a perfect fit for the wound in three-dimensional space.

To determine whether CINPs@NSFG with ROS scavenging capacity could accelerate diabetic wound healing, we evaluated its therapeutic efficiency on a full-thickness diabetic wound model (Fig. 4C). After 5 days of treatment, the ROS content in the wound bed was tested using a dedicated ROS kit to investigate the in vivo ROS scavenging capacity. As shown in Fig. 4D, E, the red fluorescent signal observed in the wounds treated by CINPs@NSFG was significantly lower than that of the control, 3 M (commercial Tegaderm dressing), and NSFG-treated groups. The results indicated that NSFG-doped CINPs could effectively scavenge ROS to relieve oxidative stress during diabetic wound healing. Figures 4F and S17 show representative pictures of wounds in different groups at days 0, 5, 10, and 15. Macroscopically, we found that the three treatment groups showed a significantly faster healing efficiency than the control group at all time points. In particular, the wound treated with CINPs@NSFG was almost healed at day 15. Moreover, the quantitative wound areas at different time points were measured based on wound closure traces (Fig. 4G, H). The quantification results showed that the wound area was 26.0% in the control group, 17.1% in the 3 M group, 15.7% in the NSFG group, and 7.1% in the CINPs@NSFG group. Subsequently, H&E staining on the tissue samples at day 15 assessed the therapeutic effects of different materials on diabetic wounds from a microscopic perspective (Fig. 4I, J). The results of histological analysis were consistent with the macroscopic photographs, and dense granulation tissue and newly formed epidermis were observed in the groups of 3 M, NSFG, and CINPs@NSFG. Specifically, squamous epithelium, sweat gland ducts, sebaceous glands, and hair follicles could be clearly observed in the wound challenged with CINPs@NSFG, presenting a typical tissue structure similar to healthy skin.

### Mechanism of CINPs@NSFG for Diabetic Wound Healing

**Fig. 5:**
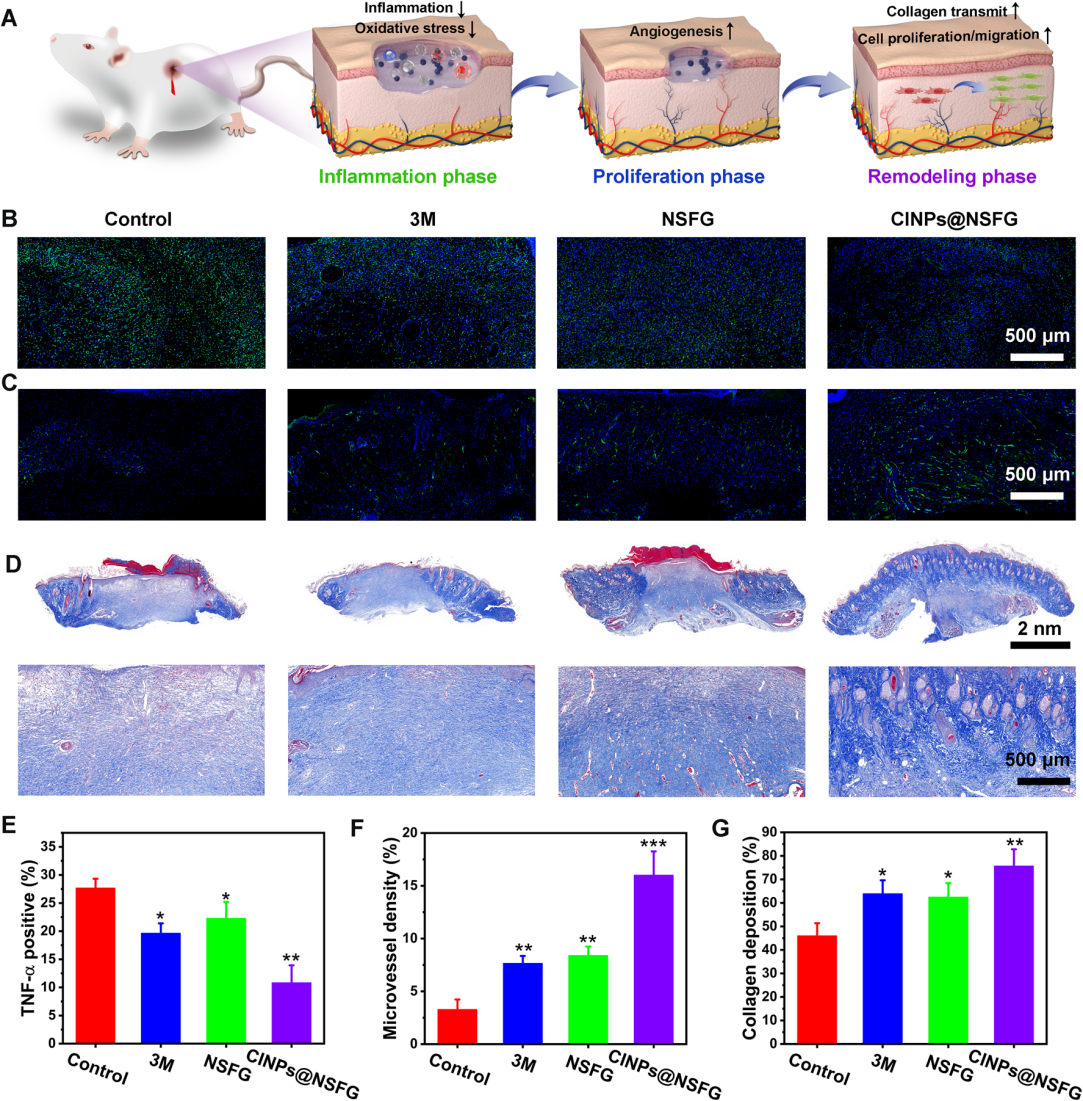
Investigating the mechanism of accelerated wound healing from the stages of inflammation, proliferation, and remodeling. **A** The proposed mechanism of CINPs@NSFG for accelerating the three phases of diabetic wound repair. **B** TNF-α staining, **C** CD31 staining, and **D** Masson’s trichrome staining of the skin tissues at different phases. Statistical analysis (*n* = 5) of images acquired from **E** TNF-α staining, **F** CD31 staining, and **G** Masson’s trichrome staining.. The bar graphs represent mean ± SD; *p* < 0.05 (*); *p* < 0.01 (**); *p* < 0.001 (***)

As an adaptive wound dressing, NSFG can cover the wound and provide a temporary barrier against external infection. Moreover, its good water absorption and moisturizing properties can promote the absorption of wound exudate and maintain a moist environment to guide skin cell reorganization and subsequent realize the infiltration and integration of host tissues [36]. Furthermore, CINPs-dotted NSFG helps to scavenge excessive ROS in diabetic wounds toward prevent oxidative stress [37]. By reducing the damage of ROS to nucleic acid, growth factors, endogenous stem cells, etc., and preventing the induction of severe inflammatory reactions, a microenvironment conducive to wound healing is finally formed [3]. The repair of skin wounds is a dynamic and highly conserved process that mainly involves three sequential and overlapping phases: inflammation, proliferation, and remodeling [38]. We then evaluated the effects of CINPs@NSFG on the three stages of wound repair (Fig. 5A). The inflammatory phase begins within hours of injury and is prolonged or even stagnant in chronic wounds. It is invariably characterized by high concentrations of proinflammatory cytokines such as tumor necrosis factor-α (TNF-α) produced by neutrophils [39]. Hence, immunofluorescent staining for TNF-α in wound tissue was performed on the fifth day. In comparison with the control group, the expression of TNF-α in the experimental groups was significantly decreased, especially in the CINPs@NSFG group (Fig. 5B, E). This indicates that the wound is gradually transitioning from the inflammatory phase to the proliferation phase, and CINPs@NSFG can accelerate this process.

An important hallmark of the proliferation phase is the formation of new vascular networks by sprouting and elongating from pre-existing vessels [40]. To this end, immunofluorescence staining for platelet endothelial cell adhesion molecule-1 (CD31) in wound tissue was performed on day 10 to assess wound angiogenesis. Compared with the control group, the three treatment groups contained more CD31, and the CINPs@NSFG group exhibiting the highest level (Fig. 5C, F). The generation of a large number of new blood vessels indicates that the wound site can receive sufficient oxygen and nutrients, which is beneficial to the collagen synthesis, fibroblast proliferation, and re-epithelialization of the wound.

In the remodeling phase, the remodeling and deposition of collagen can promote wound healing and increase breaking strength, which is an important indicator for evaluating the quality of healing tissue [41]. In order to explore the content of collagen in the remodeling stage, the wound tissue was stained by Masson’s trichrome staining on day 15. Obviously, the control group showed sparse collagen (Fig. 5D, G). In contrast, CINPs@NSFG-treated wounds showed clear new hair follicles and dense collagen, suggesting that the healing tissue had returned to a state similar to healthy skin.

### Skin Bioelectronics Equipped with AgNWs@NSFG

**Fig. 6:**
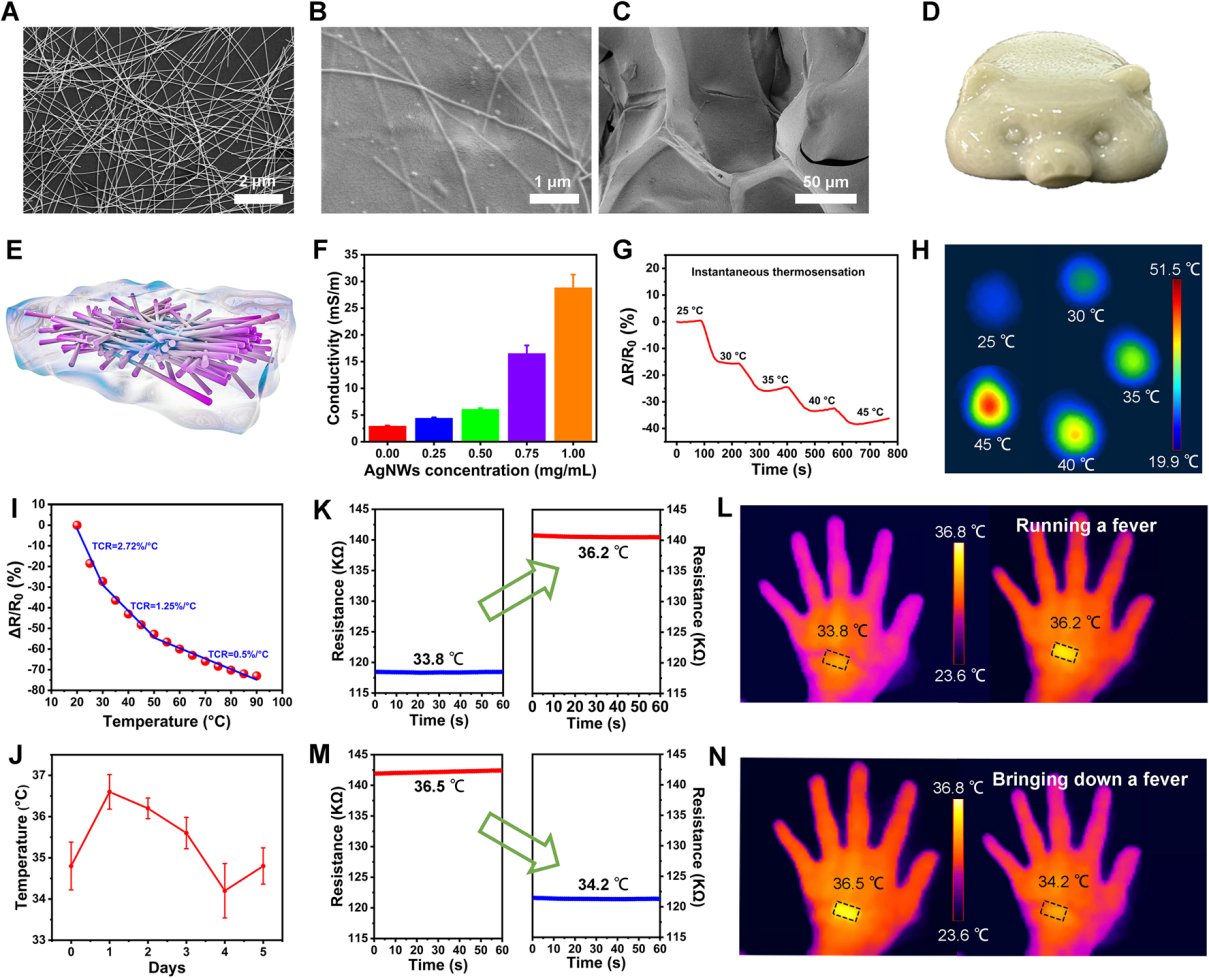
Constructing of a “fever indicator” using AgNWs@NSFG. TEM images of **A** AgNWs, **B, C** AgNWs@NSFG. **D** A piggy model casted with AgNWs@NSFG. **E**, **F** Conductivities of AgNWs@NSFG as a function of AgNWs content (n = 5). **G** Resistance changes and **H** heat distributions of AgNWs@NSFG to different temperatures. **I** Normalized relative resistance changes of AgNWs@NSFG from 20 to 90 ºC. **J** Wound temperatures recorded by AgNWs@NSFG. **K** Resistance variation and **L** infrared thermal images of the temperature sensor before and after “artificial fever.” **M** Resistance variation and **N** infrared thermal images of the temperature sensor during the process of bringing down a fever. The bar graphs represent mean ± SD

Recent advances in skin bioelectronics, such as wearable temperature sensor, soft electrodes for recording ECG and EMG signals, have enabled electrophysiological signals of the wounded to be real-time monitored and modulated [42, 43]. NSFG exhibits mechanical softness similar to human skin and can replace rigid components in conventional bioelectronics. To improve the electrical properties of NSFG, AgNWs were suspended in NSFG solution, and with a heating/cooling cycle, they form soft and electrically conductive percolating networks in the composite hydrogel (i.e., AgNWs@NSFG) (Figs. 6A–D and S18). To demonstrate its potential in soft bioelectronics, we use AgNWs@NSFG as a “fever indicator” to instantaneously indicate the wounded’s temperature and as a soft electrode to monitor ECG and EMG signals.

Firstly, the effect of AgNWs content on the conductivity of NSFG was investigated. As shown in Fig. 6E, the conductivity of AgNWs@NSFG showed an increasing trend with the increased doping of AgNWs. This is attributed to the fact that the increased content of high-aspect-ratio AgNWs promotes the formation of a continuous conductive filler network and reduces the spacing between nanowires, resulting in more hopping sites for electron transfer [44]. The thermosensitive properties of AgNWs@NSFG were also explored in the heating stage. As shown in Fig. 6F, the resistance changes in a step-like manner, indicating the rapid thermal sensitivity of AgNWs@NSFG as a temperature sensor. As shown in Fig. 6G, H, the resistance changes stepwise until a new equilibrium is achieved through heat conduction from the object to its surroundings. The temperature coefficient of resistance (TCR) is an index commonly used to evaluate the thermal sensitivity of temperature sensors and quantified as TCR = [(*R*_t_ − *R*_0_)/*R*_0_]/*δ*_T_, in which *R*_t_ represents the real-time resistance [45]. Figure 6I clearly demonstrates the negative thermosensitivity of AgNWs@NSFG temperature sensor and shows a highly linear thermal response signal within the designed operating temperature range (30 − 50 ℃). The TCR value (1.25%/℃) of our sensor over the operating temperature range exceeds most previously reported temperature sensors, indicating its superior thermal response performance. The appreciable electrical temperature-dependent trait was attributed to the tunneling conduction and carriers hopping between AgNWs surfaces, which accelerates the mobility of charge carriers at higher temperature, leading to an increase in electrical conductivity [46]. Meanwhile, this temperature sensor showed high repeatability and stability under alternating cold/hot temperatures (Fig. S19). In order to explore the influence of water evaporation on the resistance of AgNWs@NSFG, we measured its relative resistance changes at 37 °C for 48 h. As shown in Fig. S20, the resistance of AgNWs@NSFG decreased by 21.2% due to the reduction of moisture, which is obviously unacceptable for the application of temperature sensing measurement. Aiming at the thorny problem that all hydrogel sensors cannot avoid, we covered AgNWs@NSFG with a waterproof film (Fig. S19B) and tested its relative resistance change at 37 °C. The results showed that the waterproof film can prevent the water evaporation of AgNWs@NSFG at body temperature, which is reflected by an extremely low and acceptable relative resistance change. Therefore, for the water loss of resistive hydrogel sensor at body temperature, necessary packaging measures can effectively ensure the accuracy of the sensor.

A substantial increase in body temperature is not only an important sign of wound infection and inflammation, but also affects a series of chemical and enzymatic reactions in the process of wound repair [47]. Here, we demonstrated AgNWs@NSFG in monitoring temperature variations during wound healing (Fig. 6J). The results showed that the temperature of the wound site increased significantly in the previous two days, mainly because white blood cells were attracted to the injury site during the inflammatory phase, causing the wound to overheat. The temperature of the wound showed a downward trend and gradually returned to normal levels. Subsequently, we mounted AgNWs@NSFG on a healthy adult male’s hand to quantitatively reveal the temperature of human skin as a “fever indicator.” We guided the heat source closer/away from the AgNWs@NSFG to change its temperature and recorded the temperature with an infrared thermal imager. As illustrated in Fig. 6K, the sensor shows a resistance of about 118.43 kΩ, and this corresponds to the normal temperature of subject’s body surface (33.8 ºC). In contrast, if the response resistance increases rapidly to about 140.5 kΩ, this means that the temperature of body surface rises to 36.2 °C. The highly resolvable resistance response corresponding to a narrow body surface temperature range provides a strong guarantee for the promising "fever indicator" application of AgNWs@NSFG. In addition, this temperature sensor can also monitor the process of bringing down a fever through repeatable and precise resistance changes (Fig. 6M). The corresponding infrared thermal images also corroborate the reliable thermal response (Fig. 6L, N).

Being used as a “fever indicator” for the wounded, soft AgNWs@NSFG can also be assembled into a multifunctional skin sensor to detect the wounded’s ECG for reflecting their physiological state and to sensitively monitor their EMG for rehabilitation training.

**Fig. 7:**
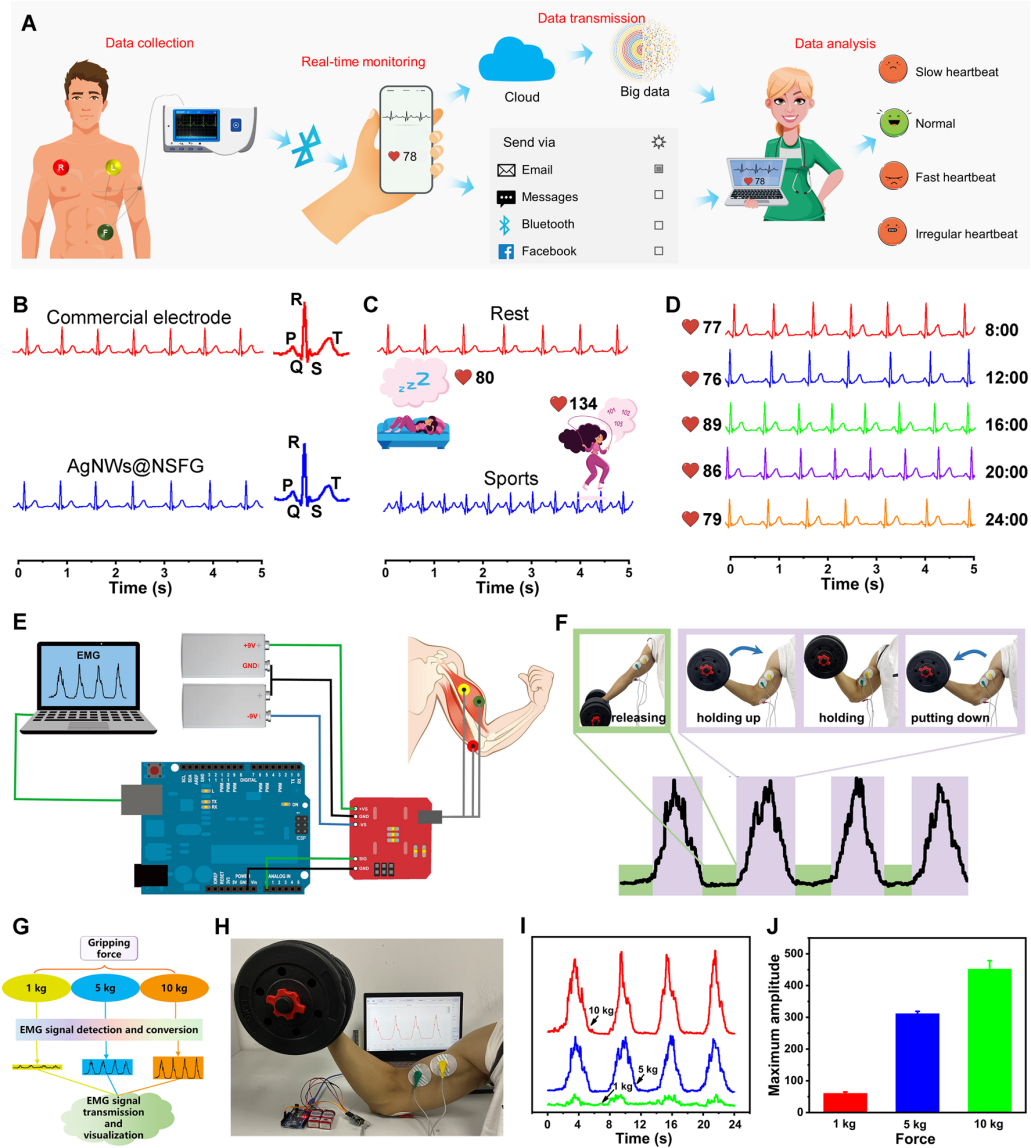
Skin sensors using AgNWs@NSFG electrodes for the detection of ECG and EMG signals. **A** Schematic illustration of the ECG detection. **B** Comparison of ECG signals of commercial electrode and AgNWs@NSFG electrode. **C** ECG signals of AgNWs@NSFG electrodes during rest and exercise. **D** Long-term monitoring of ECG signals using AgNWs@NSFG electrodes. **E** Schematic setup of EMG device. **F** EMG signal and the corresponding images of dumbbell lifting. **G** Experimental flowchart and **H** photographs of the EMG device used to measure bioelectricity of bicipital muscle. **I** EMG signals and **J** corresponding peaks value after. The bar graphs represent mean ± SD

As for recording ECG signals, a wireless ECG monitoring system was configured by adhering three AgNWs@NSFG electrodes onto the volunteers’ skin and linking them to a wireless ECG recorder (Fig. 7A). After signal collection and data processing, the ECG signal is wirelessly transmitted to a smartphone with a Bluetooth module, and a dynamic heart rate graph can be synchronously displayed on the APP (Fig. S21). Personal ECG data could be sent to the doctor through the Cloud, Email and other means for immediate clinical diagnosis, and the corresponding analysis results will be fed back: slow heartbeat, normal, fast heartbeat, and irregular heartbeat (Fig. S22). Thanks to the advantages of excellent conductivity and softness, AgNWs@NSFG electrodes obtained high-quality ECG signals, which are almost identical to that of standard commercial electrodes (Figs. 7B and S23). The amplified signals measured by two types of electrodes show the same representative ECG cycle: a clearly distinguishable P wave caused by atrial depolarization, a QRS complex representing ventricular depolarization, and a T wave reflecting ventricular repolarization [8]. AgNWs@NSFG electrodes can accurately monitor ECG signals at heart rates of 70 and 120 bpm during rest and exercise, revealing their superior performance (Fig. 7C). More importantly, they can be used for long-term healthcare monitoring, showing high-quality ECG signals throughout a day of continuous use (Fig. 7D).

EMG is a weak current generated by muscle contraction, which can be used to analyze human motion mechanics and muscle conditions, thereby assisting the wounded in rehabilitation training. As shown in Fig. 7E, an EMG monitoring platform was developed by attaching a set of AgNWs@NSFG electrodes to the arm and linking them to an Arduino-based recorder. The biopotentials of the hand muscles were collected by special software in which the signal was full-wave rectified and quantized by the root mean square method to convert the analog signal into a more recognizable digital signal (Fig. S24). As a proof of concept, EMG signals generated by arm muscles were monitored by lifting dumbbells of different weights (Figs. 7G and S25). As shown in Fig. 7F, H, the clear, concise, and positive EMG signals corresponding to the four consecutive actions of releasing, holding up, holding, and putting down the arm were successfully obtained, indicating that changes in muscle activity caused by continuous contraction can be recorded. In addition, the EMG signals enhanced with the increased gripping strength, which is the result of superposition of muscle cell action potentials (Fig. 7I, J).

## Conclusions

In summary, we introduce a kind of broadly applicable food biopolymer-based biogel that unites the challenging needs of elastic yet injectable wound dressing and skin bioelectronics in a single platform. We merge all the favorable attributes (ROS scavenging capacity and electrical conductivity) in one material that is food-safe constituents, derived entirely from natural, and has excellent modifiability, injectability, and flexibility. This is the first demonstration of a hydrogel dressing that satisfies both deep and superficial wounds and for the accelerated healing of diabetic wounds. Our biogels can be fabricated into a flexible skin bioelectronic and integrated sensing system, which can serve as a “fever indicator” and monitoring human activities and tiny electrophysiological signals (such as ECG and EMG), providing important clinical information for the rehabilitation training of the wounded. Our versatile and adaptive biogel platform represents a practical, environmentally benign, inexpensive route for future healthcare technologies that assists wound management through optimizing wound healing modalities and providing richer electrophysiological signals.

### Supplementary Information

Below is the link to the electronic supplementary material.

Supplementary file1 (PDF 1815 kb)
