# Supplementary material for: Tailoring Food Biopolymers into Biogels for Regenerative Wound Healing and Versatile Skin Bioelectronics
Source: Nanomicro Lett. 2023 Jun 7;15:153. doi: 10.1007/s40820-023-01099-1 (PMC10247910; doi:10.1007/s40820-023-01099-1)
Supplement: Supplementary file 1 — Supplementary file1 (PDF 1815 kb) [file 40820_2023_1099_MOESM1_ESM.pdf]

Supporting Information for

Tailoring Food Biopolymers into Biogels for Regenerative Wound Healing and Versatile Skin Bioelectronics

Qiankun Zeng[1], Qiwen Peng[1], Fangbing Wang[1], Guoyue Shi[1], Hossam Haick[2,] * and Min Zhang[1,] *

[1]School of Chemistry and Molecular Engineering, Shanghai Key Laboratory for Urban Ecological Processes and Eco-Restoration, East China Normal University, 200241 Shanghai, P. R. China

[2]Department of Chemical Engineering and Russell Berrie Nanotechnology Institute, Technion - Israel Institute of Technology, 320003 Haifa, Israel

*Corresponding authors. E-mail: mzhang@chem.ecnu.edu.cn (M. Zhang); hhossam@technion.ac.il (H. Haick)

S1 Experimental

S1.1 Materials

Konjac glucomannan, xanthan gum, FeCl3, FeSO4, NaCl, hydrogen peroxide (H2O2, 30%), salicylic acid, AgNO3, polyvinyl pyrrolidone (PVP), ethylene glycol were purchased from Aladdin (Shanghai, China). 2,2-diphenyl-1-picrylhydrazyl (DPPH), treptozotocin (STZ), 2',7'-dichlorofluorescein diacetate (DCFH-DA) were ordered from Sigma-Aldrich. Cell Counting Kit-8 (CCK-8) assay, Dulbecco's modified Eagle medium (DMEM), fetal bovine serum (FBS), ROS kit, PTIO• were purchased from Gibco (Thermo Fisher Scientific). All reagents and materials were all analytical purity and were used as received without purification.

S1.2 Water Absorption and Retention Properties of Biogels

At 37°C, several biogels were tested for their water absorption (WA) and water retention (WR) using the gravimetric technique. In a nutshell, a certain mass of biogel (W_0) was first swollen in PBS, and then taken out for weighing at a predetermined time point (W_t). The WA of biogels was calculated by the following equation: WR= $W_t/W_0\times100\%$. To evaluate the water retention properties of the prepared biogel, a certain mass of swollen biogel (W_s) was put into an electric thermostatic drying oven set to 37°C, and they were weighed at regular intervals. The WR was derived as follows: WR = $W_r/W_s\times100\%$. All experiments were repeated three times.

S1.3 Mechanical Performance Tests

Biogels were made into cylinder samples in preparation for the compression test (20 mm in height and 15 mm in diameter). All biogel samples were placed on the sample table of a universal tensile machine (Instron 5967, USA) and compressed at a rate of 60

nm/min until they break. For rheological test, dynamic frequency sweep (0.1–10 Hz) of cylindrical biogel samples (3 mm in height and 25 mm in diameter) was carried out on a commercial rheometer (TA Instruments, DHR-2). The test was conducted at room temperature and the fixed strain was 1%.

S1.4 Cytotoxicity Studies

The mouse Fibroblasts Cells (L929) were used to test the cytotoxicity of biogel by the Living/Dead cell double staining kit and cell counting kit 8 (CCK8) assay. Briefly, L929 cells were seeded in a 24-well plate with DMEM culture medium and cultured in the 5% CO_2 incubator at 37 ℃ for 12 h. NSFG, CINPs@NSFG, and AgNWs@NSFG were placed in transwell inserts, with the untreated group as control. After 48 hours of co-culture of biogels with cells, a Living/Dead cell double staining kit was used to evaluate the cytotoxicity of the samples. Calcine-AM, which has green fluorescence, was used to label live cells, while PI, which has red fluorescence, was used to stain dead cells. To evaluate the cell proliferation of the L929 co-cultured with various biogels, we performed the CCK8 assay. The transwell inserts were removed after 24, 48, and 72 h, and CCK8 reagents were then introduced for an hour. The cell viability was evaluated by measuring the absorbance of CCK8 at 450 nm.

S1.5 Hemolysis Assays

A hemolysis activity experiment was conducted to evaluate the blood compatibility of NSFG, CINPs@NSFG, and AgNWs@NSFG. The rat blood was centrifuged at 1000 rpm for 5 min to separate the erythrocytes. The obtained erythrocytes were diluted to a final concentration of 5% (v/v) after being washed three times with PBS. Thereafter, 1mL of diluted erythrocytes suspension was mixed with 200 mg biogel samples in a tube and shaken (150 rpm) in a 37 ℃ incubator for 1 h. To determine the level of hemolysis, the experiment also included a positive control (water) and a negative control (PBS). After centrifuging all sample tubes at 1000 rpm for 5 min, 100 μL of the supernatant was taken and placed in a 96-well plate to measure the absorbance at 540 nm by microplate reader (Infinite M200 Pro, Tecan, Austria).

S1.6 In Vivo Toxicity Evaluation

A syringe with a 23-gauge needle was used to subcutaneously inject 0.4 mL of NSFG, CINPs@NSFG, and AgNWs@NSFG into a rat back to test the substance's toxicity. The rats were sacrificed after 10 d, and histological examination (H&E staining) of the major organs (heart, liver, spleen, lung, and kidney) was carried out to assess any potential organ damage. At the same time, blood samples were taken simultaneously for the examination of the biochemical index. As a control, rat that received no treatment were employed.

S1.7 ROS Scavenging Capacity of CINPs

(1) •OH scavenging efficiency. The ·OH generated by the Fenton reaction reacts with salicylic acid to generate 2,3-dihydroxybenzoic acid with special absorption at 510 nm. The •OH scavenging efficiency of CINPs was determined by measuring the absorbance

values of salicylic acid. Herein, 100 μL of CINPs solutions, 100 μL of $FeSO_4$ (6 mM), and 200 μL of H_2O_2 (6 mM) were mixed and shaken (1000 rpm) in a 37 °C incubator for 10 min. Subsequently, 100 μL of salicylic acid (6 mM, dissolved in ethanol) was added to the above solution, followed by continued shaking for 30 minutes. After centrifuging all sample tubes at 10000 rpm for 5 min, 100 μL of the supernatant was taken and placed in a 96-well plate to measure the absorption curve at 350-700 nm, and then calculate the absorbance at 510 nm. (2) DPPH elimination evaluation. A working solution (1 mL) containing DPPH (0.125 mg/mL) and gradient concentrations of CINPs (0.1-1 mg/mL) was placed in a 1.5 mL centrifuge tube, and they were then left to react in the dark for 20 min. The absorbance values at 520 nm were then calculated from the entire wavenumber scanning curves that had been acquired. (3) PTIO• scavenging assay. A working solution (1 mL) containing PTIO• (0.15 mg/L) and gradient concentrations of CINPs (0.1-1 mg/mL) was shaken (1000 rpm) in a 37 °C incubator for 1 h. After centrifuging all sample tubes at 10000 rpm for 5 min, 200 μL of the supernatant was taken and placed in a 96-well plate to measure the absorption curve at 350-700 nm, and the absorbance values at 557 nm were recorded.

S1.8 Intracellular ROS Scavenging

L929 cells were seeded in 24-well plate containing 1 mL of culture medium per well for 12 h. After removing the culture medium, cells were cultured with DMEM containing FBS (10%), H_2O_2 (100 μM), and samples for another 12 h. Groups without H_2O_2 and samples served as negative and positive controls, respectively. After culturing for 12 h, the culture medium and biogel were removed, and the cells were washed three times with PBS. After that, 500 μL of DCFH-DA (10 μM in DMEM medium) was added into each well and incubated for 20 min. The cells were rinsed three times with serum-free media after being incubated in the dark for 20 min. After washing three times with serum-free medium, intracellular ROS levels were measured using an inverted fluorescence microscope (BDS400, CNOPTEC).

S1.9 Criteria for Distinguishing Deep from Superficial Wounds

Generally speaking, superficial wounds refer to wounds with damage to the epidermis and part of the dermis, such as skin abrasions, ulcers, second-degree burns, and blisters. Deep wounds refer to wounds that extend from the epidermis and dermis to subcutaneous tissue, fascia, and muscle damage, such as surgical wounds, third-degree burns, and incision wounds, with a depth greater than 1 cm. In this experiment, rat superficial wounds (10 mm diameter, 2 mm thickness) were created by using a circular punch. As for deep wounds, we used a scalpel to cut the muscle tissue of pork to form wounds with a thickness of about 2 cm and shapes of circles, triangles and ovals.

S1.10 *In Vivo* Diabetic Wound Healing

Male Sprague-Dawley (SD) rats weighing 200 g were purchased from Shanghai SLAC Laboratory Animal Co., Ltd. They were kept in an SPF environment at room temperature (25°C) with a 12-hour light/dark cycle and unrestricted access to food and water. After three days of dietary acclimation, rats were induced to develop type I

diabetes by intraperitoneal injection of streptozotocin (STZ) at a dose of 60 mg/kg. One week after injection, the rats with symptoms of polyphagia, polydipsia, and polyuria, and the blood glucose concentration greater than 16.7 mmol/L were designated as diabetic rats. After anesthesia, the rat's northern hair was shaved and a 10-mm full-thickness skin wound was created using a 10-mm round punch. All rats were randomly divided into four groups: blank group (control), 3M TegadermTM CHG (commercial dressing), NSFG, CINPs@NSFG groups. Wounds were photographed on days 0, 5, 10, and 15 of treatment and the area was calculated by Image J. The wound tissue was collected and fixed with paraformaldehyde (4%) for paraffin embedding. The embedded tissue was cut into 8-10 μm thick sections and attached to glass slides for subsequent histological analysis. The wound closure area was measured using hematoxylin and eosin (H&E) staining. Collagen deposition was measured by Masson's trichrome staining. To evaluate the wound healing phase, immunohistochemical labeling of ROS, TNF-α, CD31, and VEGF were performed at various time points.

S1.11 Temperature Measurements

The resistance variation of the AgNWs@NSFG sensor in response to temperature was measured by a digital multimeter (Owon B41T+) with a Bluetooth module and recorded by a mobile phone. The measured temperature of the sensor is controlled using a water bath with precise temperature control. Human hand temperature was recorded in real-time using an infrared camera (MobIR Air, Guide).

S1.12 Electrocardiography (ECG) Signal Measurement

AgNWs@NSFG was prepared as soft electrodes for electrocardiography signal detection. AgNWs@NSFG was cut into cylinders with a diameter of 1.5 cm and a thickness of 0.1 cm through a mold, and fixed to the body with electrode pads. To compare the performance of AgNWs@NSFG electrode and commercial electrodes, a commercial Ag/AgCl electrode was adopted as a control. The electrode connection method adopts the typical lead I: the positive electrode (yellow) is placed on the lower border of left clavicle, the negative electrode (red) is placed on the lower border of right clavicle, and the irrelevant electrode (green) is placed under the xiphoid process. The electrical signals sensed by AgNWs@NSFG electrodes were recorded in real time by an Easy ECG Monitor (Prince 180B, Heal Force) and wirelessly transmitted to the screen of the mobile phone via Bluetooth. Finally, the electrocardiography curves can be transmitted to the computer for statistics and analysis, and an analysis report can be generated for clinical reference.

S1.13 Electromyography (EMG) Signal Measurement

We captured EMG signals using AgNWs@NSFG as bioelectrodes to assess the effectiveness of bioelectric transmission at tissue-electrode interfaces. Two AgNWs@NSFG electrodes were attached to the biceps brachii of the right arm, and the other was attached to the elbow, serving as three sets of electrodes for EMG signal monitoring. Then lift different weights of dumbbells with hand to stimulate the

production of EMG signals. The EMG signal was captured and smoothed by the EMG sensor, and finally transmitted to the computer through the microprocessor (Arduino).

S1.14 Statistical Analysis

Results were expressed as mean ± standard deviation (SD) from at least three independent experiments. One-way analysis of variance with Tukey's test (Origin 8.0) was used for statistical analysis. The significance of differences was treated as follows: $p > 0.05$ (NS: not significant); $p < 0.05$ (*); $p < 0.01$ (**); $p < 0.001$ (***).

S2 Supplementary Figures

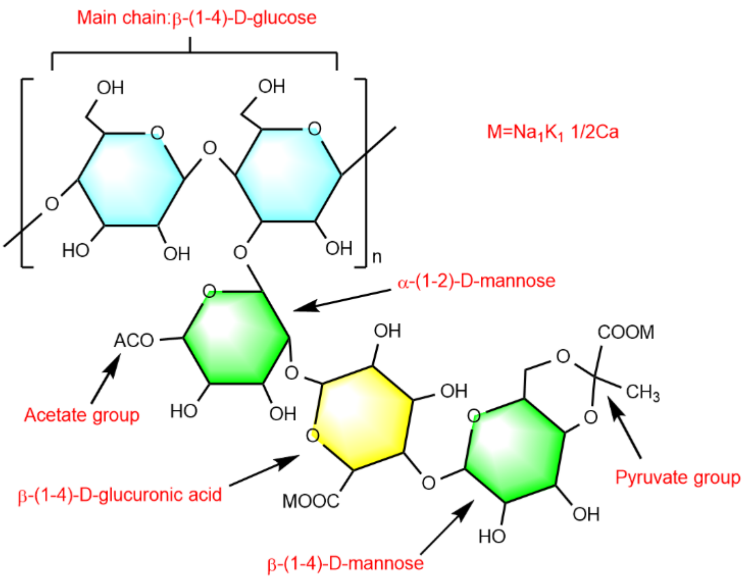

Fig. S1 Chemical structure of xanthan gum

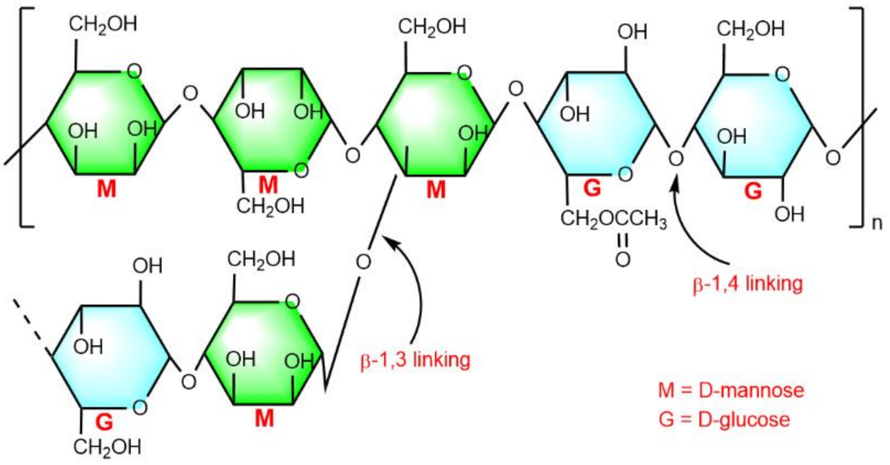

Fig. S2 Chemical structure of konjac glucomannan

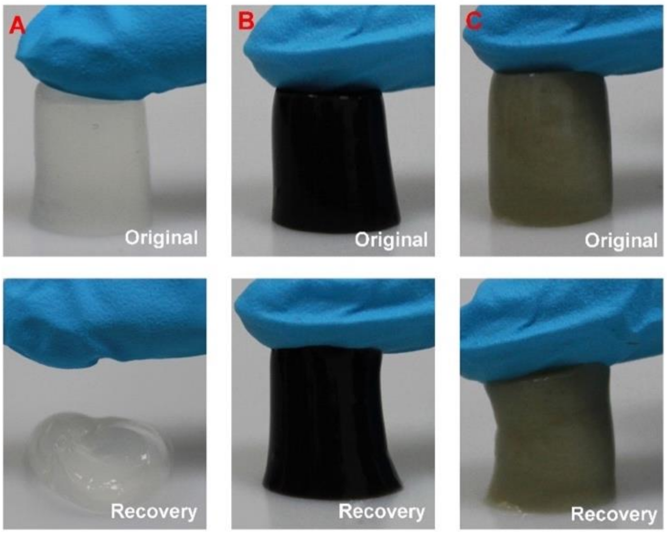

Fig. S3 Photos of the NSFG (A), CINPs@NSFG (B), and AgNWs@NSFG (C) at original and recovery

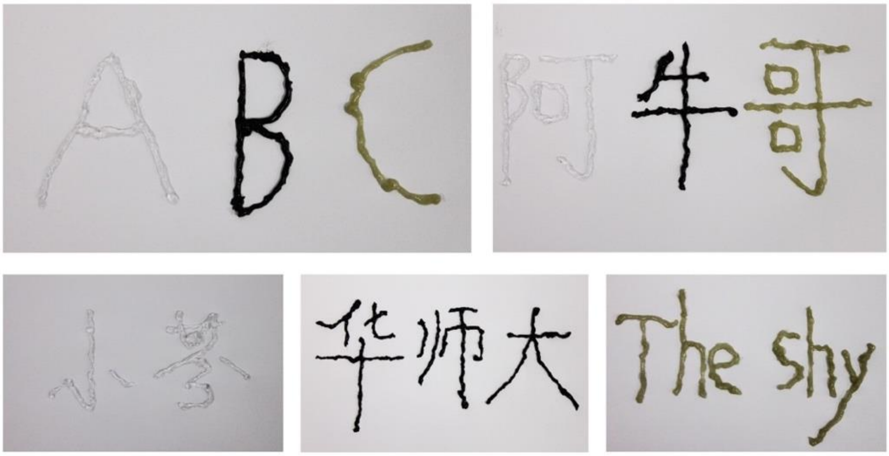

Fig. S4 The injectability of NSFG (white), CINPs@NSFG (black), and AgNWs@NSFG (yellow) through the syringe

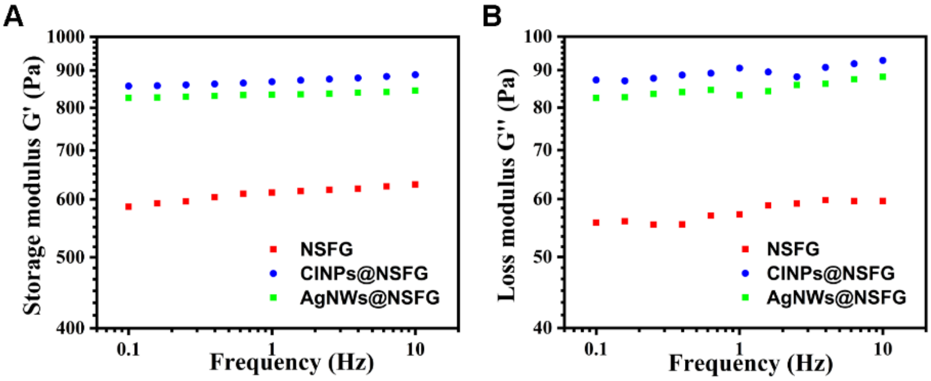

Fig. S5 Storage modulus G' (A) and loss modulus G'' (B) of different biogels

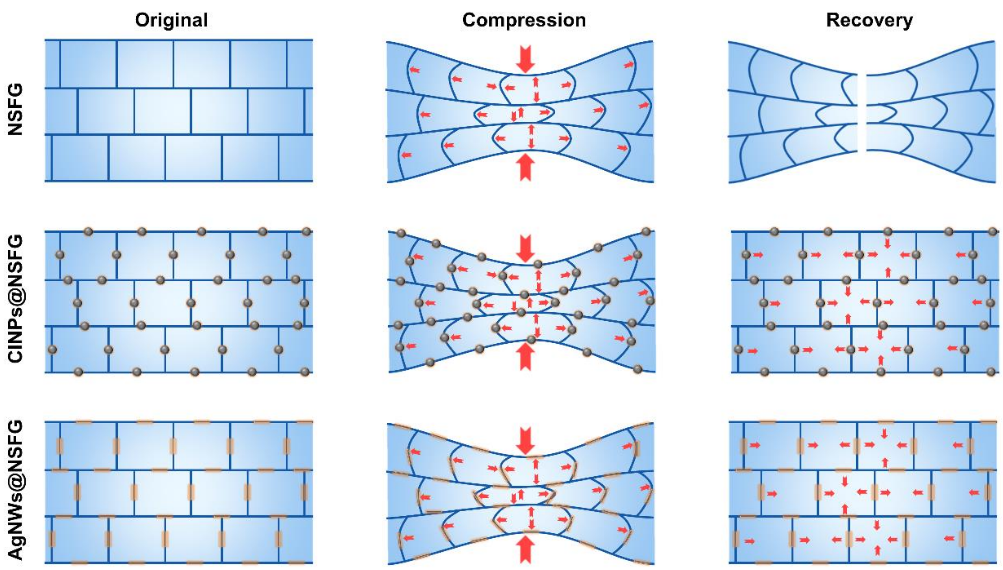

Fig. S6 Schematic diagram of the microscopic mechanism of biogels during original, compression and recovery

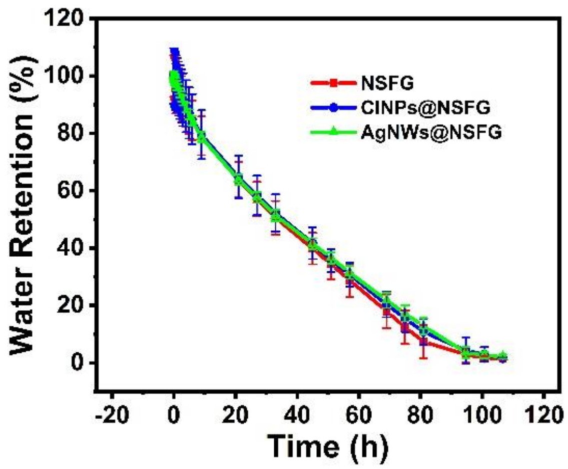

Fig. S7 Water retention property of biogels (n=3)

The change of wound pH value can reflect many physiological and biochemical processes in the wound healing process. Thus, phenol red (PR, a nontoxic dye sensitive to pH from 5 to 8) was incorporated into NSFG to form a biogel of PR@NSFG toward the monitoring of wound pH. As shown in **Fig.** S8A, the color of PR@NSFG gradually changed from yellow to red as the pH increased. The colorimetric information of PR@NSFG consists of red (R), green (G), and blue (B), which can be split into RGB (**Fig.** S8B). The results indicated that the green signal of PR@NSFG decreased with the increase of pH value and showed an excellent linear relationship (**Fig.** S8C). As PR@NSFG exhibited a good pH responsiveness in vitro, we subsequently tested this property in rat wounds. The initial pH of rat wounds was 5.5 and reached a peak of 7.6 within two days, which may be caused by a mild infection/inflammatory reaction at the wound site (**Fig.** S8D). After the third day, the pH of the wound site dropped and gradually reached normal levels.

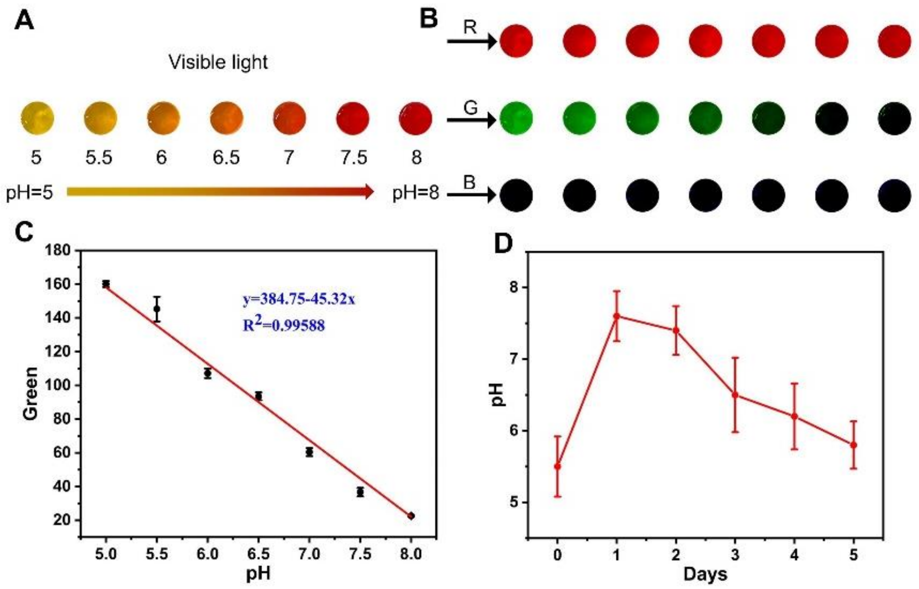

Fig. S8 (**A**) Complete color picture and (**B**) RGB images of PR@NSFG at different pH values. (**C**) A linear plot of the green intensity of PR@NSFG as a function of pH values. (**D**) pH variation of rat wound indicated from PR@NSFG

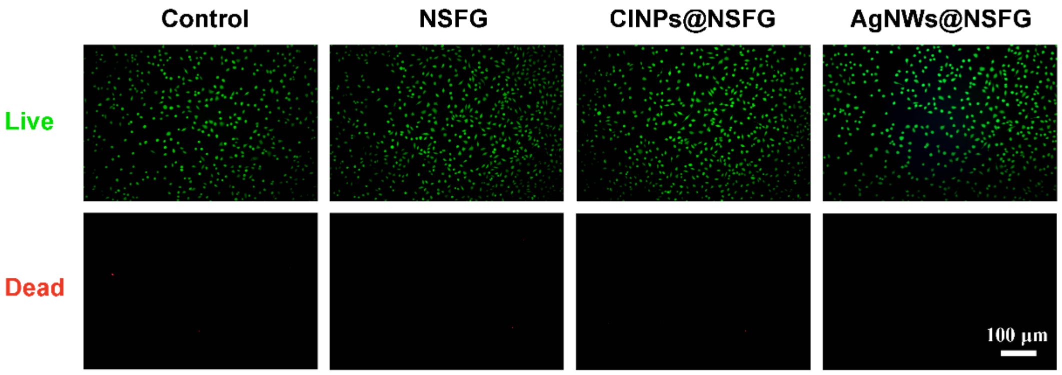

Fig. S9 Live/dead staining images of L929 after co-culture with various biogels

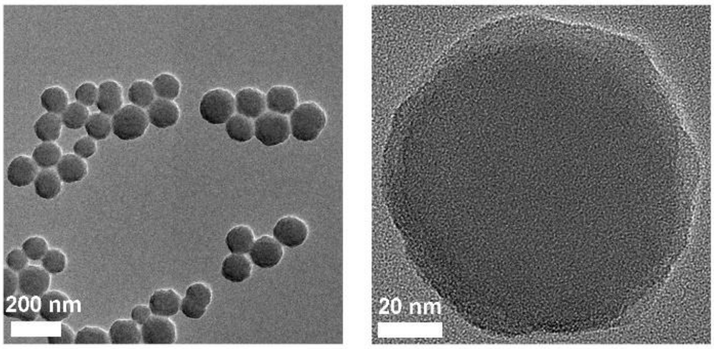

Fig. S10 TEM images of CINPs

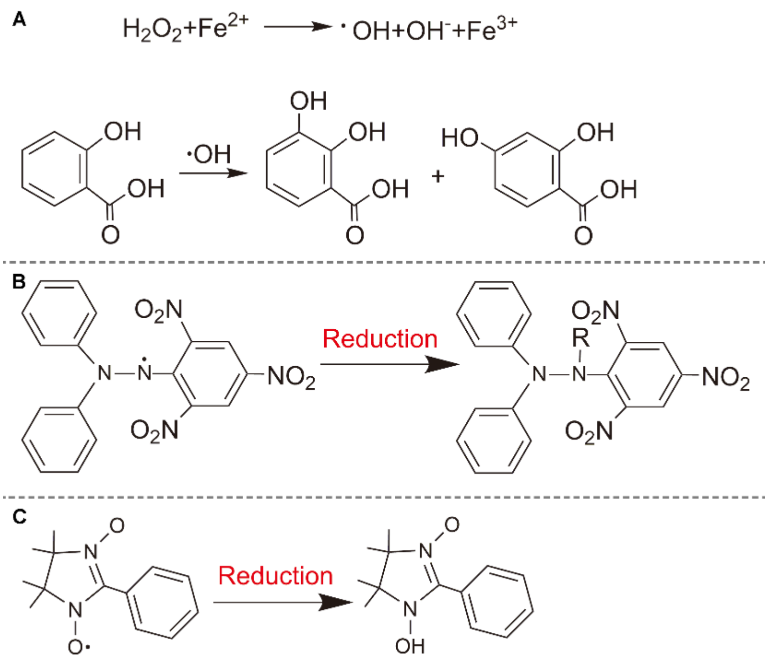

Fig. S11 Mechanism of •OH (**A**), DPPH (**B**), and PTIO (**C**) scavenging assays

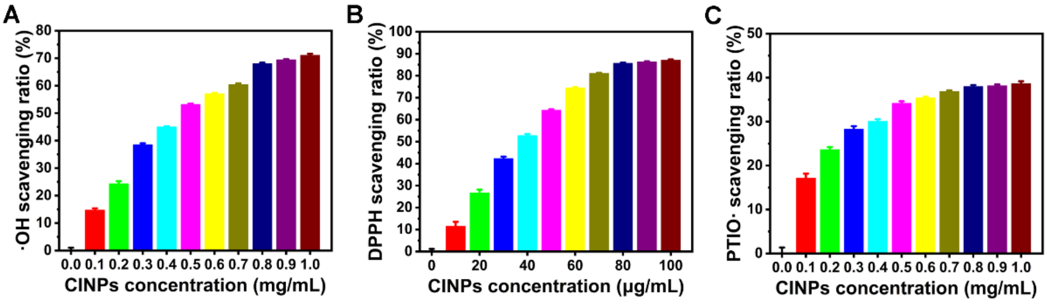

Fig. S12 The scavenging ratios of •OH (**A**), DPPH (**B**), and PTIO• (**C**) by CINPs at different concentrations (n=3)

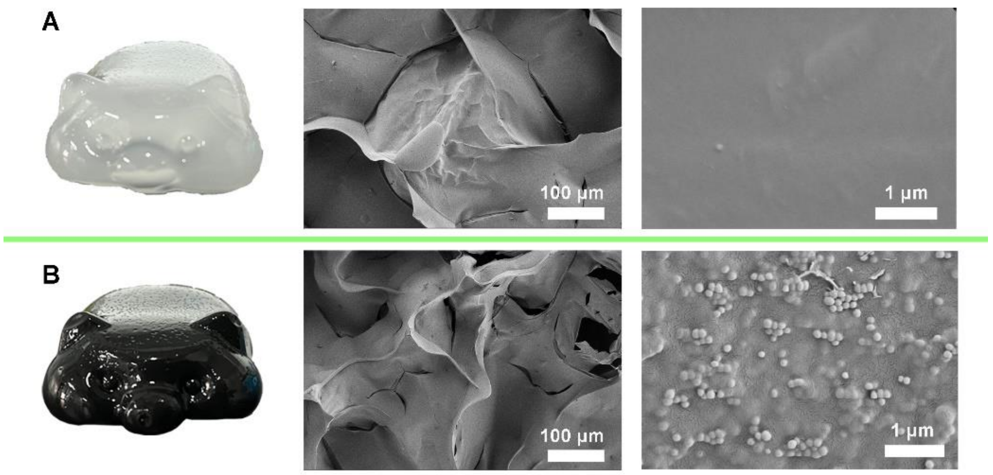

Fig. S13 Piggy molds and SEM images of (**A**) NSFG and (**B**) CINPs@NSFG

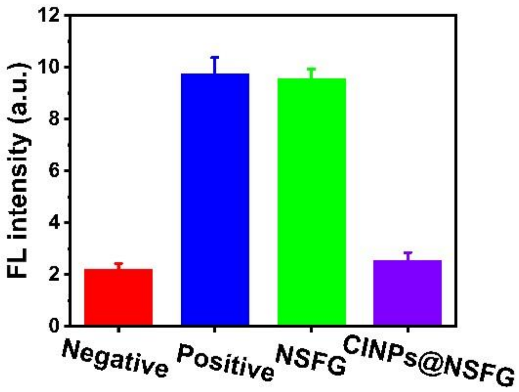

Fig. S14 Quantification of the DCFH-DA fluorescence intensity extracted from fluorescence images (n=5)

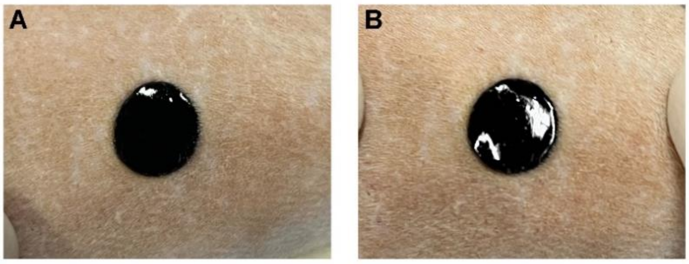

Fig. S15 Photographs of wounds containing CINPs@NSFG before (**A**) and after (**B**) being squeezed and stretched

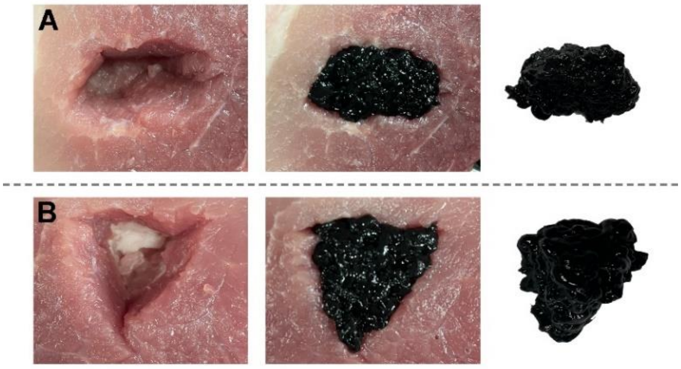

Fig. S16 Photos of deep wounds, wounds filled with CINPs@NSFG, and detached CINPs@NSFG

Both CINPs and AgNWs were incorporated into NSFG to form a biogel of CINPs/AgNWs@NSFG to treat the wound in rats with diabetes. The experimental results show that CINPs@NSFG and CINPs/AgNWs@NSFG had similar treatment effects, which indicates that the addition of AgNWs cannot interfere with the wound healing process. Of course, this also verified the important role of ROS removal in accelerating the healing of diabetes wounds.

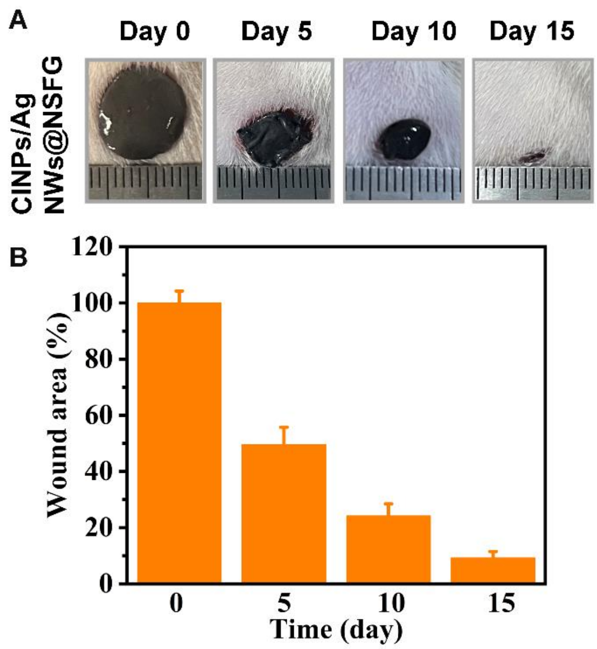

Fig. S17 Photographs (**A**) and areas statistics (**B**) of the diabetic wounds treatments with CINPs/AgNWs@NSFG at representative time points post wounding

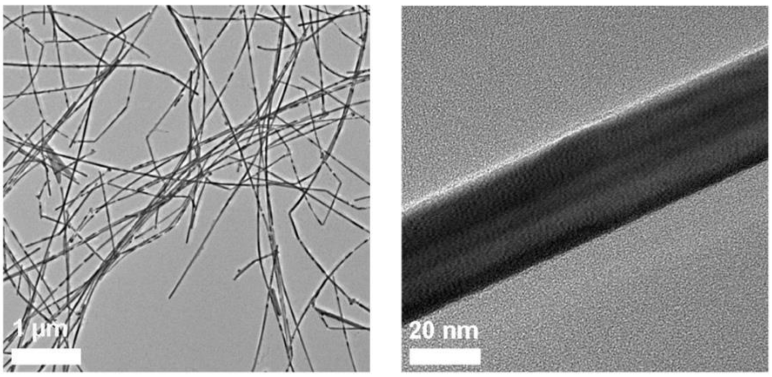

Fig. S18 TEM images of AgNWs

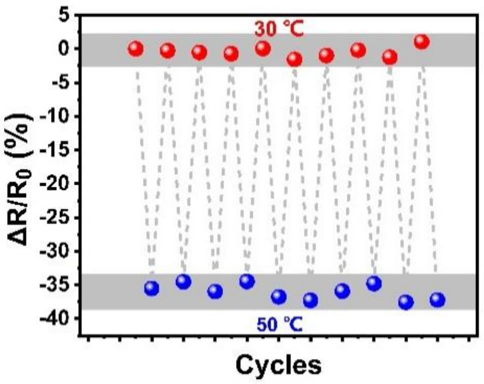

Fig. S19 The repeated temperature discrimination ability of the sensor in the process of repeated cooling (30 ℃) and heating (50 ℃)

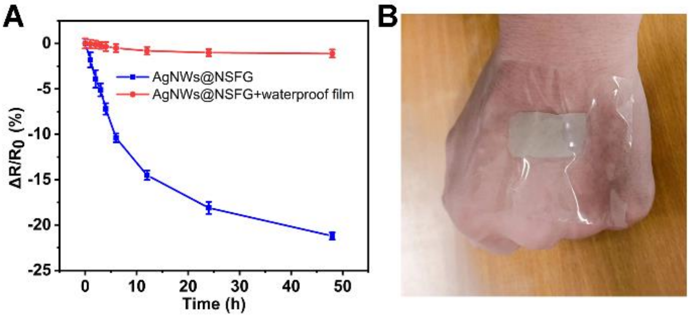

Fig. S20 (**A**) The resistance change of AgNWs@NSFG after different treatments at 37 ℃. (**B**) Photograph of AgNWs@NSFG covering with a waterproof film

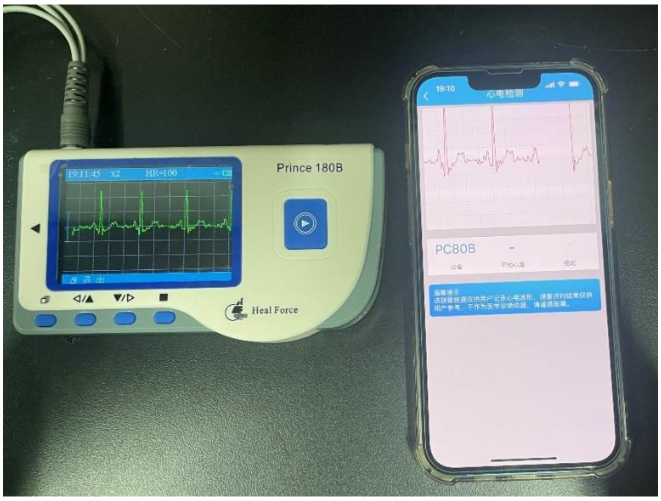

Fig. S21 Photograph showing the wireless ECG recorder and the dynamic heart rate graph displayed on the APP

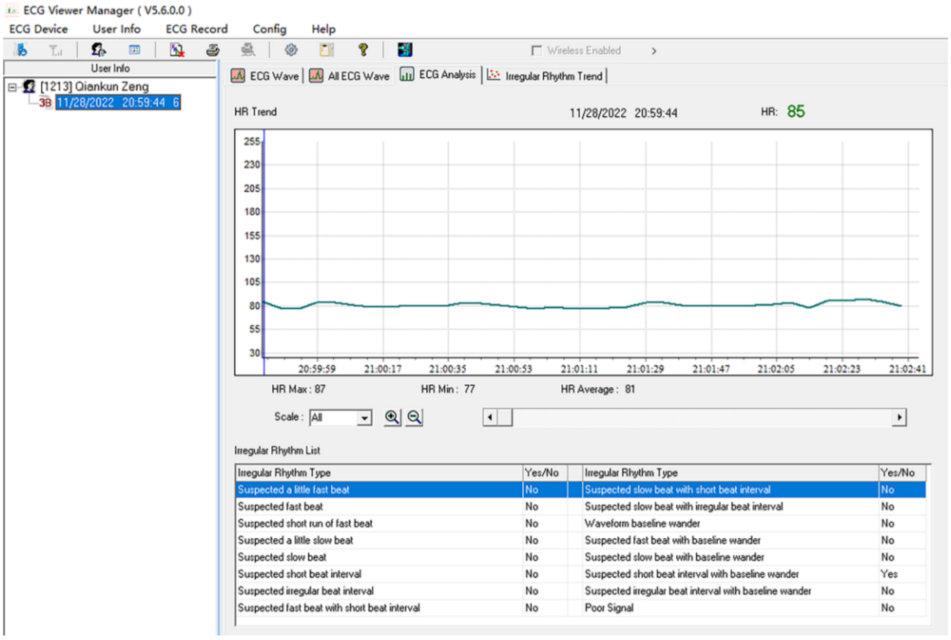

Fig. S22 The analysis results generated by doctors based on personal ECG data

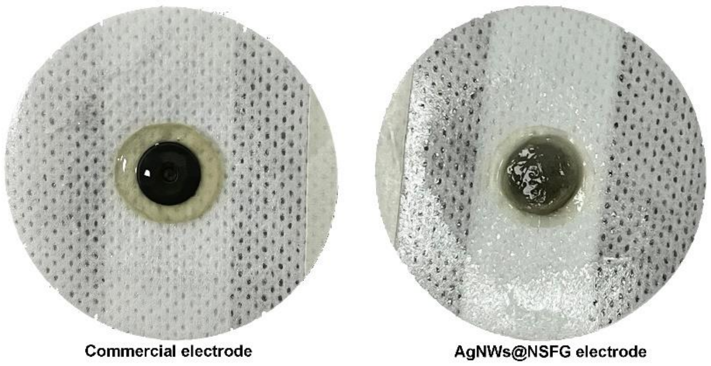

Fig. S23 Photos of commercial electrode and AgNWs@NSFG electrode

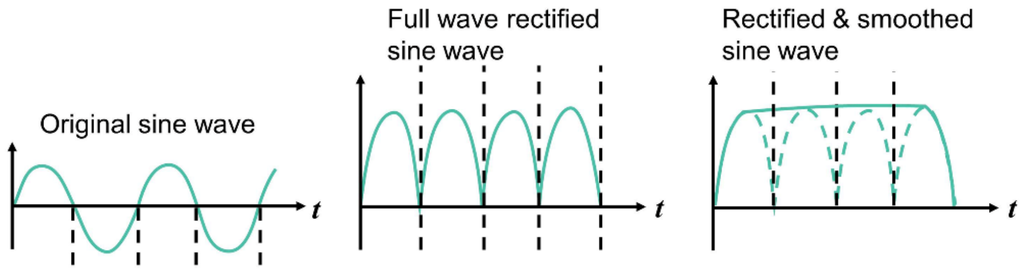

Fig. S24 EMG signal was full-wave rectified and quantized by the root mean square method: using a simple sine wave as an example

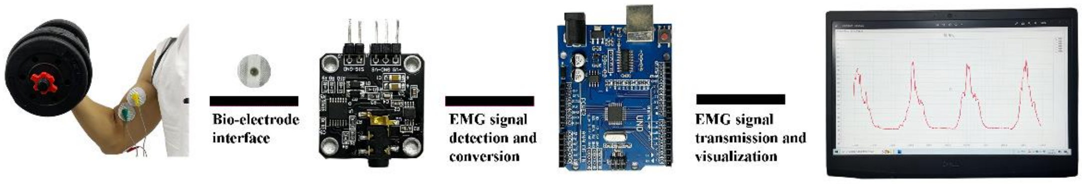

Fig. S25 Four functional units in the EMG test system

Table1 Summary of thermo-sensitivity of temperature sensors

Sensing materials	Sensing ranges (°C)	TCR (%/°C)	Refs.
Pt	0–70	0.024	[S1]
PEDOT:PSS	30−45	0.03	[S2]
reduced graphene oxide	30−80	0.9	[S3]
Ag nanocrystal/PDMS	30−50	0.185	[S4]
Graphene-MPPU	20−100	0.815	[S5]
Pt/silk fibroin	20−60	0.205	[S6]
graphene	25−50	0.06	[S7]
AgNWs@NSFG	30−50	1.25	This work

Supplementary References

[S1] Q. Hua, J. Sun, H. Liu, R. Bao, R. Yu et al., Skin-inspired highly stretchable and conformable matrix networks for multifunctional sensing. Nat. Commun. **9**, 244 (2018). https://doi.org/s41467-017-02685-9

[S2] W. An, S. Heo, S. Ji, F. Bien, U. Park et al., Transparent and flexible fingerprint sensor array with multiplexed detection of tactile pressure and skin temperature. Nat. Commun. **9**, 2458 (2018). https://doi.org/s41467-018-04906-1

[S3] Q. Trung, S. Ramasundaram, U. Hwang, E. Lee et al., An all-elastomeric transparent and stretchable temperature sensor for body-attachable wearable electronics. Adv. Mater. **28**, 502–509 (2016). https://doi.org/10.1002/adma.201504441

[S4] J. Bang, S. Lee, B. Park, H. Joh, K. Woo et al., Highly sensitive temperature sensor: ligand-treated Ag nanocrystal thin films on PDMS with thermal expansion strategy. Adv. Funct. Mater. **29**, 1903047 (2019). https://doi.org/10.1002/adfm.201903047

[S5] X. Hu, M. Tian, T. Xu, X. Sun, B. Sun et al., Multiscale disordered porous fibers for self-sensing and self-cooling integrated smart sportswear. ACS Nano **14**, 559–567 (2019). https://doi.org/10.1021/acsnano.9b06899

[S6] J. Huang, Z. Xu, W. Qiu, F. Chen, Z. Meng et al., Stretchable and heat-resistant protein-based electronic skin for human thermoregulation. Adv. Funct. Mater. **30**, 1910547 (2020). https://doi.org/10.1002/adfm.201910547

[S7] Y. Yang, Y. Song, X. Bo, J. Min, S. Pak et al., A laser-engraved wearable sensor for sensitive detection of uric acid and tyrosine in sweat. Nat. Biotechnol. **38**, 217–224 (2020). https://doi.org/s41587-019-0321-x
